# Utilization of colorectal cancer screening tests: a systematic review and time trend analysis of nationally representative data

**DOI:** 10.1016/j.eclinm.2024.102783

**Published:** 2024-08-21

**Authors:** Idris Ola, Rafael Cardoso, Michael Hoffmeister, Hermann Brenner

**Affiliations:** a Division of Clinical Epidemiology and Aging Research, German Cancer Research Center (DKFZ), 69120, Heidelberg, Germany; b Medical Faculty Heidelberg, University of Heidelberg, 69120, Heidelberg, Germany; c Division of Preventive Oncology, German Cancer Research Center (DKFZ) and National Center for Tumor Diseases (NCT), 69120, Heidelberg, Germany; d German Cancer Consortium (DKTK), German Cancer Research Center (DKFZ), 69120, Heidelberg, Germany

**Keywords:** Colorectal cancer, Cancer screening, Fecal tests, Endoscopy, Cancer prevention

## Abstract

**Background:**

The substantial and increasing global burden of colorectal cancer (CRC) underscores the imperative to enhance implementation and utilization of effective CRC screening offers. Therefore, we examined the lifetime and up-to-date use of CRC screening tests across various countries, and described utilization trends over time.

**Methods:**

We conducted a systematic review on the extent and recent trends of utilization of CRC screening tests among people 45 years or older in different countries around the globe. PubMed/Medline, Web of Science, and Embase electronic databases were screened for eligible studies from inception to June 30, 2024. The study protocol was registered with international prospective register of systematic reviews (PROSPERO) (CRD42023391344).

**Findings:**

A total of 50 studies, based on nationally-representative data, were finally included - 27 from the United States (US) and 23 from other countries. The overall utilization of CRC screening has steadily increased over time in many countries, reaching 74.9% in Denmark in 2018–2020, 64% in Korea in 2020, and 72% in the US in 2021. Nevertheless, the utilization rates remain far below the national or continental targets in most countries. In contrast to European and Asian countries, where screening was predominantly fecal test-based, the approach in the US was primarily driven by colonoscopy, and the uptake of fecal tests and sigmoidoscopy gradually declined in the past two decades.

**Interpretation:**

Despite ongoing progress in CRC screening offers and utilization, there remains large potential for enhanced roll-out and utilization of effective CRC screening programs for enhanced control of CRC incidence and mortality in the years ahead.

**Funding:**

There was no funding source for this study.

**Research in context**

**Evidence before this study**

Despite ample evidence demonstrating the efficacy of diverse colorectal cancer (CRC) screening strategies in reducing CRC incidence and mortality, substantial disparities persist, encompassing the adoption of screening tests by the population, the initiation and types of population screening programs, test availability, and ultimately, utilization rates worldwide.

In addition to manually searching the reference lists of relevant published articles, comprehensive literature searches were conducted in PubMed, Web of Science, and Embase from inception to June 2024. Search terms including ‘colorectal cancer, or neoplasms, or carcinoma,’ were combined with terms such as ‘fecal tests,’ ‘fecal occult blood test,’ ‘fecal immunochemical test,’ ‘colonoscopy,’ ‘endoscopy,’ and ‘sigmoidoscopy,’ as well as terms denoting screening strategies such as ‘mass screening,’ ‘early detection of cancer,’ ‘population-based screening,’ or ‘community-based screening.’ Our literature search showed that while many countries still fall short of achieving their screening utilization targets, the ongoing evolution and growing adoption of CRC screening guidelines underscore the need for timely documentation of the impact of screening policies and practice decisions over time. In this systematic review, we examined the lifetime and up-to-date utilization of CRC screening tests globally and investigated usage trends over time.

**Added value of this study**

Our review provides comprehensive documentation and analysis of CRC screening test utilization rates spanning the last four decades. It highlights the impacts and significance of screening test availability, preventive health policies, and the diverse screening guidelines and methodologies implemented across various countries and regions on screening test utilization among eligible populations across the world.

**Implications of all the available evidence**

In light of the recent development of new guidelines, such as those set forth by the American College of Gastroenterology, alongside ongoing evaluations of existing screening programs, such as the European programs, and the growing adoption of population-based CRC screening beyond Europe and North America, our review offers timely insights with practical and policy implications. These insights have the potential to influence the future trajectory of CRC screening programs, aiming to meet the increasing need to reduce CRC incidence and mortality.

## Introduction

Colorectal cancer (CRC) persists as a disease of paramount global health significance, constituting over one-tenth of the global cancer burden and leading to approximately one million cancer-related fatalities each year.^1^

Nevertheless, the slow development and the long latent phase of the disease and the availability of efficient screening methods provide great opportunities to lower the burden of CRC by population-based CRC screening.^2^ Specifically, fecal tests, flexible sigmoidoscopy (FS) and colonoscopy are screening modalities for which the potential to considerably reduce CRC incidence and mortality has been demonstrated by a large body of observational epidemiological studies and randomized trials.^3^, ^4^, ^5^, ^6^ However, these screening tests have either been unavailable or insufficiently utilized in many countries around the world.^7^^,^^8^ There are also substantial regional variations in the availability and use of these tests, which are largely influenced by the type of screening offer, nature of the testing procedure, accessibility, and personal attributes of eligible individuals.^8^, ^9^, ^10^, ^11^

To facilitate screening uptake, many countries and regions set various time-bound population CRC screening utilization and offering targets, e.g. reaching 80% utilization by 2018 in the US and offering screening to 90% of eligible individuals by 2025 in Europe.^12^^,^^13^

With the development of new screening modalities and changes in the epidemiology of CRC, several technical guidelines and recommendations have been made and updated over the years,^13^^,^^14^ including introduction of computed tomographic (CT) colonography, fecal immunochemical test (FIT), fecal DNA tests in combination with a FIT (mt-sDNA), and commencement of screening at younger age of 45 years.^14^^,^^15^ Other guidelines, especially in Europe, have also placed emphasis on the screening framework, prioritizing organized systems over an opportunistic approach.^13^

Given these continuous changes, it is important to monitor screening utilization to evaluate progress over time and identify possible barriers, enhancers, and disparities to utilization inherent in various screening methods and systems. In this systematic review, we mapped the lifetime and up-to-date use of CRC screening tests across various countries, and investigated trends over time.

## Methods

### Study design, protocol and registration

A systematic review of the literature on the utilization of CRC screening tests across the world was undertaken. The protocol for this study was registered with international prospective register of systematic reviews (PROSPERO) in January 2023 (CRD42023391344) (https://www.crd.york.ac.uk/PROSPERO/).

### Data sources and search strategy

The electronic databases of PubMed/Medline, Web of Science, and Embase were searched for relevant articles using relevant search terms, including Medical Subject Headings in PubMed/Medline. The search terms include “colorectal cancer”, “fecal tests”, “fecal immunochemical test”, “colonoscopy”, endoscopy”, “sigmoidoscopy”, “screening”, population-based screening, and early detection of cancer. The full combination terms and search results from electronic databases are presented in Table S1.

Searches were conducted in January 23-March 16, 2023, covering the period from inception until December 2022, with no restrictions applied. Cross-referencing by manual review of the reference lists of included articles was performed to add to the final list of included articles. A repeat search was conducted to identify new articles published up to June 30, 2024.

### Study selection

All citations were imported into Rayyan QCRI systematic review software.^16^ Titles, abstracts, and full-texts of articles were independently screened by two reviewers (IO, RC) according to eligibility criteria per protocol. The preferred reporting items for systematic reviews and meta-analyses (PRISMA) guidelines for reporting of systematic reviews were followed.

**Fig. 1:**
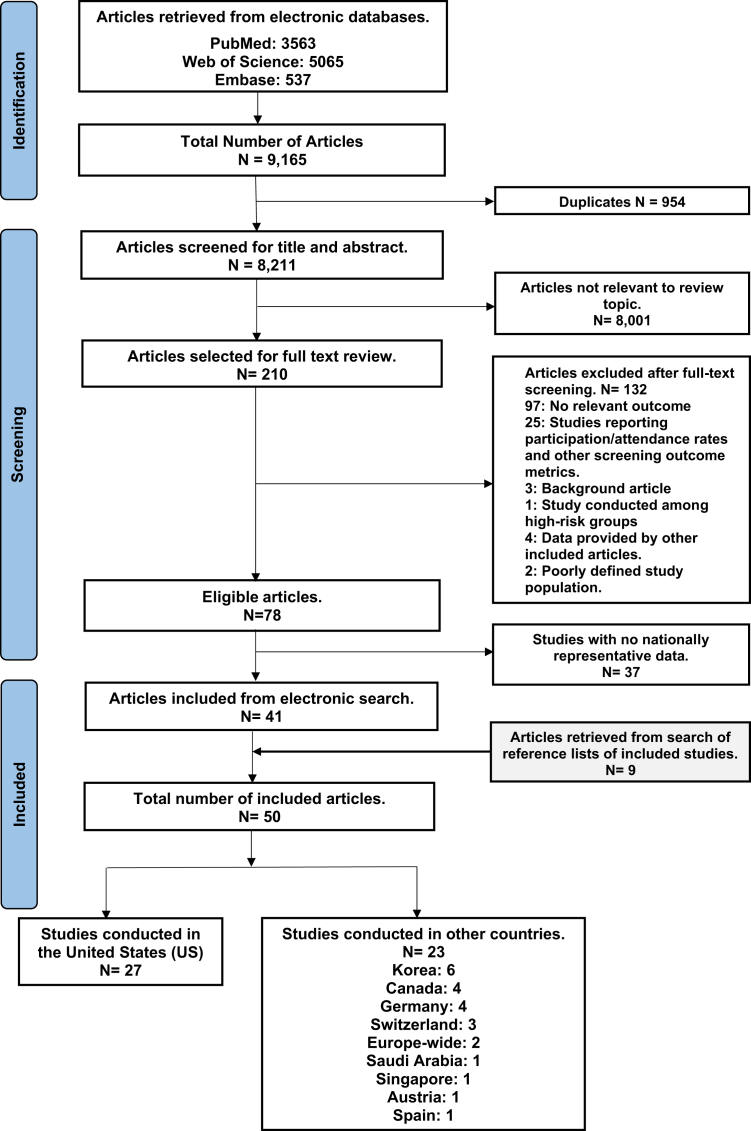
Flowchart of the article selection process for the review.

Relevant studies that presented national estimates of CRC screening test utilization in the eligible population aged 45 years and older were included in the review. Studies that focused primarily on CRC screening among higher-risk populations, such as those with a family history of CRC, Lynch syndrome, and inflammatory bowel disorders, were excluded. Studies that reported CRC screening use as a follow-up procedure after positive screenings only or CRC treatment were not considered as were those with small sample sizes fewer than 150.^7^ The data extraction flow chart is presented in Fig. 1, and the list of excluded articles, with reasons and references, is presented in Supplementary appendix 2, Table E1 and Item E2.

### Data extraction and quality assessment

Two reviewers (IO and RC) independently and manually extracted data from the included studies using a pretested form in Microsoft® Excel. Information extracted included study characteristics: demographic information of study participants; outcomes studied; and the utilization rate of CRC screening tests.

Quality assessment and risk of bias were independently assessed by two reviewers (IO and RC) using the Effective Public Health Practice Project (EPHPP) assessment tool for observational studies.^17^ Discrepancies were resolved by discussion between the two reviewers and, where necessary, additional co-authors (HB and MH) to resolve disagreements.

### Outcomes

The outcome of interest was the national estimates of utilization of CRC screening tests according to relevant guidelines. Broadly, this involves utilization of guaiac-based fecal occult blood test (gFOBT) or FIT within the last 1–2 years, mt-sDNA within the past 3 years, flexible sigmoidoscopy and CT colonography within the last 5 years, and colonoscopy or endoscopy within the last 5–10 years.

### Data synthesis and analysis

Study outcomes were descriptively summarized and results presented by type of CRC screening tests. Unless otherwise specified, weighted proportions were used as reported in studies that used large population-based samples with complex sampling strategy. When reported, the corresponding confidence intervals, as well as age- and sex-specific estimates, were also presented. Due to the disproportionately large number of eligible studies from the US, analysis by type of CRC tests was sub-grouped by location, mainly the US and all other countries. A time trend analysis for utilization of each test was descriptively conducted using studies with consistent outcome definitions. For many countries, we were unable to look at temporal trends in screening utilization because data for several time points was lacking.

All figures (except Fig. 1) were produced using the “ggplot2” package in RStudio Version 4.3.2 (RStudio, Inc., Boston, MA, USA).

### Statistics

No statistical tests were applied in the study.

### Ethics statement

Patient consent for publication: Not applicable.

### Role of the funding source

There was no funding source for this study.

## Results

### Data sources and quality ratings

From an initial pool of 9165 articles retrieved and screened based on title and abstract, 210 underwent full-text screening. Ultimately, 50 observational studies, offering nationally-representative estimates, were included in the final review spanning data from 1987 to 2021 (Fig. 1). For each EPHPP domain, the quality ratings for the included studies were between low and moderate, essentially due to the self-reported nature of the data (Supplementary appendix 1, Table S2).

Twenty-seven studies presented screening estimates for the US, predominantly using population-based data from the National Health Interview Survey (NHIS) and the Behavioral Risk Factor Surveillance System (BRFSS).^18^, ^19^, ^20^, ^21^, ^22^, ^23^, ^24^, ^25^, ^26^, ^27^, ^28^, ^29^, ^30^, ^31^, ^32^, ^33^, ^34^, ^35^, ^36^, ^37^, ^38^, ^39^, ^40^, ^41^, ^42^, ^43^, ^44^ Additionally, six studies reported estimates for Korea,^45^, ^46^, ^47^, ^48^,^50^ four each for Germany,^51^, ^52^, ^53^,^54^ and Canada,^55^, ^56^,^58^ three for Switzerland,^59^, ^60^, ^61^ and one each for Singapore,^62^ Saudi Arabia,^63^ Austria,^64^ and Spain.^65^ Furthermore, reports by Cardoso et al.^8^ and Ola et al.^10^ provided estimates for 30 European countries based on data from the European Health Interview Survey (EHIS) in 2013–2015 and 2018–2020, respectively.

### Fecal tests use in the US

Nineteen studies provided estimates for fecal tests (gFOBT or FIT) in the US.^18^, ^19^, ^20^, ^21^, ^22^, ^23^, ^24^, ^25^, ^26^, ^27^, ^28^, ^29^, ^30^, ^31^, ^32^, ^33^, ^34^, ^35^, ^36^ (Table 1) Recent use of fecal tests, mainly defined as use within 1–3 years, was reported in all studies except Zhu et al.,^34^ with estimates ranging from 27.1% in 1998 to 12.6% in 2020. The usage of fecal tests among the newly eligible age group of 45–49 was estimated at 2.4% in 2021.^36^ In addition, recent use of mt-sDNA ranged between 2.7% in 2018 and 5.8% in 2020^33^^,^^35^ (Table 1, Table S3).

**Table 1:** Estimates of recent and lifetime use of fecal test (gFOBT or FIT, or mt-sDNA) from the United States.^a^

First author (Yr)	Study characteristics				CRC test utilization			
	Data source	Latest year	Sample size	Age	Time frame	Overall	Men	Women
Anderson^c^ (1995)^18^	NHIS	1992	4428	50+	3 yrs	26.3%	25.4%	26.9%
					1 yr	17.3%	NR	
Breen^c^ (2001)^19^	NHIS	1998	11,925	50+	2 yrs	27.1%^b^	28.5%	26.1%
Nadel^c^ (2002)^20^	NHIS	1998	12,128	50+	1 yr	19.5% (18.7–20.3)	NR	
Swan^c^ (2003)^21^	NHIS	2000	11,622	50+	1 yr	25%^b^	24.0%	26.0%
Seef^c^ (2004)^22^	NHIS	2000	11,480	50+	1 yr	17.1% (16.3–17.9)	16.8%	17.5%
					Ever	36.7% (35.6–37.8)	NR	
Subramanian (2005)^23^	NHIS	2000	12,505	50+	1 yr	13.3%^b^	NR	
Liang^c^ (2006)^24^	NHIS	2003	11,779	50+	Ever	13.6%^b^	NR	
					Up-to-date (1 yr)	6.6%^b^		
Meissner^c^ (2006)^25^	NHIS	2003	11,548	50+	1 yr	15.6%	16.1%	15.3%
Shapiro^c^ (2008)^26^	NHIS	2005	11,918	50+	1 yr	12.0% (11.3–12.7)	12.6%	11.6%
Doubeni^c^ (2010)^27^	MCBS	2005	7614	65–80	2 yrs	10.4%	NR	
Swan^c^ (2010)^28^	NHIS	2005	11,890	50+	1 yr	12.0%^b^	12.3%	11.8%
Klabunde^c^ (2011)^29^	NHIS	2008	7776	50–75	1 yr	10.9% (10.0–12.0)	NR	
Shapiro^c^ (2012)^30^	NHIS	2010	8952	50–75	1 yr	8.8% (8.1–9.6)	8.9%	8.7%
Cole^c^ (2012)^31^	BRFSS	2005	466,175	50+	1 yr	8.8%^b^	NR	
de Moor^c^ (2018)^32^	NHIS	2015	12,541	50–75	1 yr	7.1% (6.5–7.8)	7.5%	6.8%
Shapiro^c^ (2021)^33^	NHIS	2018	10,595	50–75	gFOBT/FIT: 1 yr	8.8% (8.1–9.5)	11.7%	10.8%
					mt-sDNA: 3 yrs	2.7% (2.4–3.2)		
Zhu^c^ (2021)^34^	HH survey^d^	2019	1595	45–75	gFOBT: Ever	47.5%	43.8%	50.9%
					mt-sDNA: Ever	25.8%	24.1%	27.1%
He^c^ (2023)^35^	BRFSS	2020	175,698	50–75	gFOBT/FIT: 1 yr	12.6%	12.2%	13.0%
					mt-sDNA: 3 yrs	5.8%	5.5%	6.2%
Star^c^ (2024)^36^	NHIS	2021	NA	45–49	gFOBT/FIT: 1 yr	2.4% (1.8–3.3)	NR	

Abbreviations: NHIS, National Health Interview Survey; MCBS, Medicare Current Beneficiary Survey (MCBS) Access to Care data; BRFSS, Behavioral Risk Factor Surveillance System; HH survey, Household survey; gFOBT, guaiac-based fecal occult blood test; FIT, fecal immunochemical test; mt-sDNA, multi-target stool DNA test; NR, not reported; yr(s), year(s).

a Table ordered first according to publication year, then the year of data collection.

b Data calculated from the available information in the article.

c Other information (e.g. estimates for other years reported in the study, or estimates by age or sex) are provided in Table S3).

d Sample sizes for the subgroups are very small and may impact the reliability of the subgroup estimates.

### Endoscopy use in the US

For endoscopy utilization, 21 studies provided estimates, with test intervals of 3–5 years for sigmoidoscopy, 5–10 years for colonoscopy, and 5 years for CT colonography.^19^, ^20^, ^21^, ^22^, ^23^, ^24^, ^25^, ^26^, ^27^, ^28^, ^29^, ^30^, ^31^, ^32^, ^33^, ^34^, ^35^, ^36^, ^37^, ^38^, ^39^ (Table 2) Lifetime colonoscopy use was reported by five studies,^22^^,^^24^^,^^34^^,^^37^^,^^38^ ranging from 23% in 1997 to 72% in 2019.^34^

**Table 2:** Estimates of recent and lifetime use of lower gastrointestinal endoscopy or CT colonography from the US.^a^

First author (Yr)	Study characteristics				CRC test utilization			
	Data source	Latest year	Sample size	Age	Time frame	Overall	Men	Women
Breen^c^ (2001)^19^	NHIS	1998	11,925	50+	FS: 3 yrs	13.6%^b^	19.0%	9.8%
Nadel^c^ (2002)^20^	NHIS	1998	12,190	50+	Proc/FS: 3 yrs	13.6% (12.9–14.3)	NR	
					Proc/FS: 5 yrs	15.9% (15.1–16.7)	21.1%	11.6%
Swan^c^ (2003)^21^	NHIS	2000	11,622	50+	Endo: 3 yrs.	25.0%	NR	
Seef^c^ (2004)^22^	NHIS	2000	11,588	50+	Col: 10 yrs; FS: 5 yrs.	33.9% (32.9–35.0)	37.4%	31.1%
					Ever	38.1% (37.1–39.2)	NR	
Chao (2004)^37^	CPS II	1997	129,246	50–79	FS/Col: 5 yrs	14.2%^b^	17.1%	11.9%
					Ever	23.0%^b^	27.1%	19.6%
Subramanian^c^ (2005)^23^	NHIS	2000	12,505	50+	Col: 5 yrs	29.1%	31.8%	26.5%
					FS: 5 yrs	15.3%	16.8%	13.9%
Liang^c^ (2006)^24^	NHIS	2003	11,779	50+	Col: 10 yrs/Ever	29.1%/29.1%^b^	NR	
					FS: 5 yrs/Ever	4.2%/3.0%^b^		
Meissner^c^ (2006)^25^	NHIS	2003	11,395	50+	Col: 10 yrs	30.8%^b^	32.2%	29.8%
					FS: 5 yrs	6.6%^b^	7.6%	5.9%
Shapiro^c^ (2008)^26^	NHIS	2005	12,045	50+	Endo: 10 yrs	45.2% (44.0–46.4)	46.9%	43.9%
Doubeni^c^ (2010)^27^	MCBS	2005	23,833	65–80	Endo: 10 yrs.	<5 yrs: 48.9%; >5 yrs: 9.0%	NR	
Swan^c^ (2010)^28^	NHIS	2005	12,016	50+	Col: 10 yrs; FS: 5 yrs	21.5%^b^	23.7%	19.8%
Klabunde^c^ (2011)^29^	NHIS	2008	7776	50–75	Col: 10 yrs	47.5% (45.9–49.0)	NR	
					FS: 5 yrs	2.4% (1.9–3.0)		
Shapiro^c^ (2012)^30^	NHIS	2010	8952	50–75	Col: 10 yrs	54.6% (53.2–55.9)	54.4%	54.7%
					FS: 5 yrs	1.3% (1.0–1.6)	1.6%	1.0%
					CT Col: Ever	1.3% (1.0–1.7)	1.7%	1.0%
Cole^c^ (2012)^31^	BRFSS	2005	466,175	50+	FS: 5 yrs; Col: 10 yrs.	29.0%^b^	NR	
de Moor^c^ (2018)^32^	NHIS	2015	12,541	50–75	Col: 10 yrs	58.3% (57.0–59.6)	57.6%	59.1%
					FS: 5 yrs	0.7% (0.5–0.9)	1.0%	0.3%
Sanford (2019)^38^	NHIS	2015	83,176	50–75	Col: Ever	Current/former/never smokers 43.8%/65.2%/57.7%	NR	
Hong^c^ (2021)^39^	NHIS	2018	34,768	45–85	CT col: 5 yrs	1.3%	1.5%	1.2%
Shapiro^c^ (2021)^33^	NHIS	2018	10,595	50–75	Col: 10 yrs;	61.1% (59.9–62.3)	61.4%	60.8%
					FS: 5 yrs	2.4% (2.1–2.8)	2.7%	2.1%
					CT col: 5 yrs	1.0% (0.8–1.3)	1.2%	0.8%
Zhu^c^ (2021)^34^	HH survey	2019	1595	50–75	Col: Ever	72.2%	72.5%	72.6%
He^c^ (2023)^35^	BRFSS	2020	175,698	50–75	Col: 10 yrs	64.5%	63.3%	65.3%
					FS: 5 yrs	3.8%	4.7%	3.2%
					CT col: 5 yrs	2.7%	3.2%	2.3%
Star^c^ (2024)^36^	NHIS	2021	NA	45–49	Col: 10 yr	17.8% (16.0–19.8)	NR	

Abbreviations: NHIS, National Health Interview Survey; CPS II, Cancer Prevention Study (CPS) II Nutrition Cohort; BRFSS, Behavioral Risk Factor Surveillance System; MCBS, Medicare Current Beneficiary Survey (MCBS) Access to Care data; HH survey, Household survey; Endo, endoscopy; Col., colonoscopy; CT Col, computed tomographic colonoscopy; FS, flexible sigmoidoscopy; NR, not reported; yr(s), year(s).

a Table ordered first according to publication year, then the year of data collection.

b Data calculated from the available information in the article.

c Other information (e.g. estimates for other years reported in the study, or estimates by age or sex) are provided in Table S4).

Regarding recent use of colonoscopy, eight studies^23^, ^24^, ^25^, ^26^^,^^29^^,^^32^^,^^33^^,^^35^ reported estimates varying between 19.0% in 2000 to 64.5% in 2020 among individuals aged 50–75.^29^^,^^35^ Recent estimate indicate a 17.8% utilization rate among individuals aged 45–49 as of 2021.^36^ Concerning sigmoidoscopy use, 10 studies reported its use as a separate screening modality.^19^^,^^20^^,^^23^, ^24^, ^25^, ^26^^,^^29^^,^^30^^,^^32^^,^^33^^,^^35^ The highest use of sigmoidoscopy was reported for 2000 (15.3%); as of 2015, less than 1% of the US adult population reported recent sigmoidoscopy use.^23^^,^^32^

Seven studies reported up-to-date use of either colonoscopy or sigmoidoscopy, with estimates varying from 13% in 1987 to 45.2% in 2005.^21^^,^^22^^,^^26^, ^27^, ^28^^,^^31^^,^^37^ Although not frequently recommended, CT colonography was reported by four studies,^26^^,^^27^^,^^32^^,^^35^ with the highest estimate reaching 2.7% as of 2020.^35^ Generally, the utilization of all endoscopic screening methods increased with age, peaking around age 65–75, and was more prevalent among men than women (Table 2, Table S4).

### Combined use of fecal tests, sigmoidoscopy, and/or colonoscopy in the US

Twenty studies,^19^, ^20^, ^21^, ^22^, ^23^, ^24^, ^25^, ^26^^,^^29^, ^30^, ^31^, ^32^, ^33^^,^^35^^,^^36^^,^^40^, ^41^, ^42^, ^43^, ^44^ provided combined data on fecal test use within 1–3 years, sigmoidoscopy within 3–5 years, and/or colonoscopy within 5–10 years (Table 3). Overall, the extracted data reveals a consistent increase in CRC screening utilization over the past three decades, increasing from 23% in 1987^19^ to 72.4% in 2020.^35^ As of 2021, CRC screening test utilization among the newly eligible 45–49 age group was estimated at 19.7%.^36^ Overall, utilization was highest for ages 65–74, while sex differences in utilization yielded mixed results with no substantial variations observed (Table 3, Table S5).

**Table 3:** Estimates of use of fecal tests, FS and/or colonoscopy (combined) in the United States.^a^

First author (Yr)	Study characteristics				CRC test utilization			
	Data source	Latest year	Sample size	Age	Time frame	Overall	Men	Women
Breen^c^ (2001)^19^	NHIS	1998	11,925	50+	FOBT: 2 yrs; FS: 3 yrs.	33.0%^b^	37.1%	30.2%
Nadel^c^ (2002)^20^	NHIS	1998	12,072	50+	FOBT:1 yr; Proct/FS: 5 yrs	22.9% (22.0–23.8)	27.1%	19.6%
Swan^c^ (2003)^21^	NHIS	2000	11,622	50+	FOBT:1 yr or Endo: 3 yrs.	39.5%^b^	40.6%^b^	38.9%^b^
Seef^c^ (2004)^22^	NHIS	2000	11,468	50+	FOBT + endo.	42.5% (41.4–43.5)	44.5%	41.0%
					Ever	54.2% (53.1–55.2)	NR	
Subramanian^c^ (2005)^23^	NHIS	2000	12,505	50+	FOBT: 1 yr; FS: 5 yrs; Col. 10 yrs.	25.1%	26.7%	23.8%
Liang^c^ (2006)^24^	NHIS	2003	11,779	50+	FOBT: 1 yr; FS: 5 yrs; Col. 10 yrs.	42.0%	45.0%^b^	42.0%^b^
					Ever	55.0%	57.0%	56.0%
Meissner^c^ (2006)^25^	NHIS	2003	11,302	50+	FOBT: 1 yr; FS: 5 yrs, Col-10 yrs	44.5%^b^	46.5%	43.1%
Shapiro^c^ (2008)^26^	NHIS	2005	11,943	50+	FOBT: 1 yr; Endo: 10 yrs	50.0% (48.8–51.2)	51.7%	48.7%
Klabunde^c^ (2011)^29^	NHIS	2008	7776	50–75	Up-to-date with screening	54.5% (52.9–56.2)	NR	
Shapiro (2012)^30^	NHIS	2010	8825	50–75	FOBT: 1 yr; FS: 5 yrs, Col.: 10 yrs	58.3% (56.6–60.1)	58.2%	58.4%
Cole^c^ (2012)^31^	BRFSS	2005	466,175	50+	FOBT: 1 yr; FS: 5 yrs; Col.: 10 yrs.	52.1%^b^	NR	
Sabatino^c^ (2015)^40^	NHIS	2013	13,045	50–75	FOBT: 1 yr; FS: 5 yrs; Col: 10 yrs	57.8% (56.6–59.0)	56.7%	58.9%
White^c^ (2017)^41^	NHIS	2015	12,650	50–75	FOBT: 1 yr; FS: 5 yrs; Col: 10 yrs	62.4% (61.1–63.7)	NR	
de Moor^c^ (2018)^32^	NHIS	2015	12,541	50–75	FOBT: 1 yr; FS: 5 yrs; Col: 10 yrs	61.3% (60.0–62.6)	60.7%	62.0%
Shapiro^c^ (2021)^33^	NHIS	2018	10,595	50–75	FOBT/FIT: 1 yr Col: 10 yrs; CT col./FS: 5 yrs. mt-sDNA: 3 yrs	66.9% (65.8–68.1)	67.4	66.5%
Sokale^c^ (2022)^42^	BRFSS	2020	779,143	50–75	FOBT/FIT: 1 yr Col: 10 yrs; FS: 5 yrs + FOBT/FIT-3 yrs.	72.5% (71.9–73.1)	NR	
Santiago-Rodríguez^c^ (2023)^43^	NHIS	2018	43,624	50–75	FOBT: 1yr, FS: 5 yrs + FOBT: 3 yrs, Col: 10 yrs.	62.8% (62.2–63.3)	NR	
He^c^ (2023)^35^	BRFSS	2020	175,698	50–75	FOBT/FIT: 1 yr; FS: 5 yrs + FOBT/FIT-3 yrs; Col: 10 yrs	70.4%	69.3%	71.4%
					FIT: 1 yr; mt-sDNA: 3 yrs; FS: 5–10 yrs; CT col: 5 yrs; Col: 10 yrs;	72.4%	71.2%	73.5%
Castañeda-Avila (2024)^44^	BRFSS	2012–2020	989,700	50–75	FOBT/FIT: 1 yr; Col: 10 yrs; FS: 5 yrs + FOBT/FIT-3 yrs.	66.5%	47.7%	52.3%
Star^c^ (2024)^36^	NHIS	2021	NA	45–49	gFOBT/FIT: 1 yr; Col: 10 yrs	19.7% (17.8–21.6)	NR	

Abbreviations: NHIS, National Health Interview Survey; BRFSS, Behavioral Risk Factor Surveillance System; FOBT, fecal occult blood test; gFOBT, guaiac-based fecal occult blood test; FIT, fecal immunochemical test; mt-sDNA, multi-target stool DNA test; Endo, endoscopy; Col., colonoscopy; FS, flexible sigmoidoscopy; Proct., proctoscopy; NR, not reported; yr(s), year(s).

a Table ordered first according to publication year, then year of data collection.

b Data calculated from the available information in the article.

c Other information (e.g. estimates for other years reported in the study, or estimates by age or sex) are provided in Table S5).

### Fecal test use in other countries

All studies included from other countries provided estimates for fecal test utilization, except for two studies from Germany^53^^,^^54^ and one each from Canada^58^ and Korea.^50^ The reported estimates were for fecal tests conducted within 1–2 years, except for studies presenting data on lifetime use (Table 4).

**Table 4:** Estimates of recent and lifetime use of fecal test (gFOBT or FIT) in other countries.^a^

First author (Yr)	Study characteristics					CRC test utilization			
	Country	Data source	Latest year	Sample size	Age	Time frame	Overall	Men	Women
Cardoso^c^ (2020)^8^	30 European countries	EHIS	2015	125,375	50–74	2 yrs	By country: see Table S6	NR	
Ola^c^ (2024)^10^	29 European countries	EHIS	2020	129,750	50–74	2 yrs	By country: see Table S6	NR	
Wahidie (2023)^64^	Austria	AHIS	2019	8267	50+	Ever	80.1%^b^	NR	
Sewitch (2007)^55^	Canada	CCHS	2003	17,498	50+	2 yrs	15.1%	NR	
Major^c^ (2015)^56^	Canada	CCHS	2012	9973	50+	2 yrs	23.0% (22.0–24.0)	NR	
Singh^c^ (2015)^57^	Canada	CCHS	2012	NR	50–74	2 yrs	30.1%	30.0%	30.2%
Sieverding (2010)^51^	Germany	HCAP	2004	15,810	50–70	Ever	53.4%^b^	44.0%	62.5%^b^
Wahidie (2022)^52^	Germany	GHU	2015	11,757	50+	Ever	81.9%^b^	NR	
Choi^c^ (2010)^45^	Korea	KNCSS	2008	922	50–79	1 yr	21.3%	21.0%	21.6%
Park^c^ (2012)^46^	Korea	KNCSS	2011	4100	50+	1 yr	25.0%	NR	
Suh^c^ (2016)^47^	Korea	KNCSS	2013	4100	50+	1 yr	27.6%	NR	
Bui^c^ (2017)^48^	Korea	KNCSS	2014	2066	50–74	Ever	52.1%	50.4%	49.6%
Choi^c^ (2018)^49^	Korea	KNCSS	2013	2154	50+	1 yr	29.7%	52.8%	47.2%
Khoja (2018)^63^	Saudi Arabia	SNSEH	2007	2946	60+	1 yr	4.4%	NR	
Wong (2013)^62^	Singapore	HH survey	2008	1743	50+	1 yr	20.9%	22.4%	20.0%
Portero de la Cruz^c^ (2023)^65^	Spain	SNHS and EHS	2020	6929	50–69	2 yrs	**Overall:** 38.0% 2020: 43.9%	37.7%	38.3%
Spaeth (2013)^59^	Switzerland	SHIS	2007	NR	50+	1 yr	7.7%	NR	
Fedewa^c^ (2015)^60^	Switzerland	SHIS	2012	7224	50–75	2 yrs	9.8%	NR	
Schneider^c^ (2022)^61^	Switzerland	SHIS	2017	8038	50–75	2 yrs	5.3%	5.4%	5.2%

Abbreviations: CCHS, Canadian Community Health Survey; HH survey, Household survey; HCAP, Health Care Access Panel; KNCSS, Korean National Cancer Screening Survey; SHIS, Swiss Health Interview Survey; SNSEH, Saudi National Survey for Elderly Health; EHIS, European Health Interview Survey; AHIS, Austrian Health Interview Survey; SNHS, Spanish National Health Survey; EHS, European Health Survey; GHU, German Health Update; gFOBT, guaiac-based fecal occult blood test; NR, not reported; yr(s), year(s).

a Table ordered first according to country (in alphabetical order), then the year of data collection.

b Data calculated from the available information in the article.

c Other information (e.g. estimates for other years reported in the study, or estimates by age or sex) are provided in Table S6).

Recent fecal test utilization varied widely among countries. Data from Switzerland between 2007 and 2017 revealed a declining pattern (from 13% to 5.3%)^60^^,^^61^ similar to the pattern observed in the US. Conversely, findings from the Korean National Cancer Screening Survey indicated a rise in recent fecal test utilization, increasing from 3.8% in 2004 to 27.6% in 2013.^47^ A comparable upward trend was noted in Canada in 2003–2012 (from 15.1% to 30.1%).^55^, ^56^, ^57^ In Singapore, as of 2008, about one-fifth of the eligible population had recently utilized fecal tests.^62^ By 2012–2013, utilization had increased to cover close to one-third of the eligible population in both Canada and Korea.^47^^,^^49^^,^^57^ In Europe, utilizing data from EHIS 2013–2015 and 2018–2020, Cardoso et al.^8^ and Ola et al.^10^ provided estimates categorized by type of CRC screening offer. Utilization within the past two years ranged from 3.6% in Romania to 51.5% in France in 2013–2015^8^ and from 3.6% in Bulgaria to 67.1% in Denmark in 2018–2020,^10^ with more favorable rates overall observed in countries offering fecal tests within fully implemented national organized screening programs^8^^,^^10^ (Table S6).

Five studies, one each from Singapore,^62^ Canada,^57^ Korea,^49^ Switzerland,^61^ and Spain,^65^ presented recent utilization estimates by sex, revealing fairly equal utilization between males and females. Additionally, two studies from Canada^56^^,^^57^ and one each from Korea^49^ and Spain^65^ reported recent fecal test use by age group. Utilization generally increased with increasing age, and was highest in age group 64–74 years (Table S6).

### Endoscopy use in other countries

Nineteen studies reporting estimates on the utilization of colonoscopy or sigmoidoscopy were included These studies include one each from Singapore,^62^ Saudi Arabia,^63^ and Austria,^64^ two from Canada,^55^^,^^57^ three from Switzerland,^59^, ^60^, ^61^ four from Germany,^51^, ^52^, ^53^,^54^ five from Korea,^45^, ^46^, ^47^, ^48^, ^49^ and two from 30 European countries^8^^,^^10^ (Table 5).

**Table 5:** Estimates of recent and lifetime use of lower GI endoscopy (Colonoscopy or FS) in other countries.^a^

First author (Yr)	Study characteristics					CRC test utilization			
	Country	Data source	Latest year	Sample size	Age	Time frame	Overall	Men	Women
Cardoso^c^ (2020)^8^	30 European countries	EHIS	2015	125,375	50–74	Col: 10 yrs	By country: see Table S7	NR	
Ola^c^ (2024)^10^	29 European countries	EHIS	2020	129,750	50–74	Col: 10 yrs	By country: see Table S7	NR	
Wahidie (2023)^64^	Austria	AHIS	2019	8267	50+	Col: Ever	61.1%^b^	NR	
Sewitch (2007)^55^	Canada	CCHS	2003	17,498	50+	10 yrs	20.6%	NR	
Singh^c^ (2015)^57^	Canada	CCHS	2012	NR	50–74	FS/Col: 10 yrs	37.2%	37.6%	36.9%
Sieverding^c^ (2010)^51^	Germany	HCAP	2004	15,810	50–70	Col: Ever	35.6%	33.4%^b^	36.8%^b^
Starker^c^ (2017)^53^	Germany	GEDA/EHIS	2014/15	9489	55+	Col: 10 yrs	58.5%	60.8%	56.5%
Wahidie (2022)^52^	Germany	GHU	2014/15	11,757	50+	Col: Ever	60.1%^b^	NR	
Hornschuch^c^ (2022)^54^	Germany	GePaRD	2017	7,475,668	NR	Col: 10 yrs	31.0%	See Table S7	
Choi^c^ (2010)^45^	Korea	KNCSS	2008	922	50–79	Endo: 10 yrs	20.5%	19.3%	21.6%
Park^c^ (2012)^46^	Korea	KNCSS	2011	4100	50+	Col: 10 yrs	23.6%	NR	
Suh^c^ (2016)^53^	Korea	KNCSS	2013	4100	50+	Col: 10 yrs	35.2%	NR	
Bui^c^ (2017)^48^	Korea	KNCSS	2014	2066	50–74	Col: Ever	41.4%	52.8%^b^	47.3%^b^
Choi (2018)^49^	Korea	KNCSS	2013	2154	50+	Endo: 10 yrs	37.3%	NR	
Khoja (2018)^63^	Saudi Arabia	SNSEH	2007	2946	60+	Col: 5 yrs	0.6%	NR	
Wong (2013)^62^	Singapore	HH survey	2007/8	1743	50+	Col: 10 yrs FS: 5 yrs	Col. 14.0% Sig: 10.8%	**Col:** 16.1% **Sig:** 12.1%	**Col:** 12.7% **Sig:** 10.0%
Spaeth (2013)^59^	Switzerland	SHIS	2007	NR	50+	Endo: 5 yrs	6.4%	NR	
Fedewa^c^ (2015)^60^	Switzerland	SHIS	2012	7224	50–75	Endo: 10 yrs	15.0%	NR	
Schneider^c^ (2022)^61^	Switzerland	SHIS	2017	8038	50–75	Col: 10 yrs	42.8%	42.8%	42.7%

Abbreviations: CCHS, Canadian Community Health Survey; HH survey, Household survey; HCAP, Health Care Access Panel; KNCSS, Korean National Cancer Screening Survey; SHIS, Swiss Health Interview Survey; GEDA: Gesundheit in Deutschland aktuell; SNSEH, Saudi National Survey for Elderly Health; EHIS, European Health Interview Survey; GePaRD, German Pharmacoepidemiological Research Database; GHU, German Health Update; AHIS, Austrian Health Interview Survey; Col., colonoscopy; FS, flexible sigmoidoscopy; NR, not reported; yr(s), year(s).

a Table ordered first according to country (in alphabetical order), then the year of data collection.

b Data calculated from the available information in the article.

c Other information (e.g. estimates for other years reported in the study, or estimates by age or sex) are provided in Table S7).

Lifetime use of colonoscopy was reported by two studies from Germany,^51^^,^^52^ and one from Korea,^48^ and Austria.^64^ In Germany, estimates ranged from 35.6% in 2004 to 60.1% in 2014/2015,^51^^,^^52^ while utilization reached 61.1% in Austria.^64^

Substantial variations were noted in recent colonoscopy utilization estimates across the included countries. The lowest reported estimate was in Saudi Arabia at 0.6% in 2006–2007,^63^ while the highest utilization was reported in Germany at 58.5% in 2014.^53^ In Korea, utilization increased from 14.4% to 35.2% in 2004–2013.^47^ A comparable pattern was observed in Switzerland in 2007–2017 (21.9–42.8%).^61^ In Europe, data from both EHIS surveys indicated variations in recent colonoscopy use based on the type of CRC screening offered. Austria maintained the highest rates among 50–74-year-olds in 2013–2015 and 2018–2020 (51.6% and 54.4%, respectively), while Bulgaria equally showed the lowest utilization for both years (2.3% and 4.4%, respectively).^8^^,^^10^

Four studies reported aggregated estimates for colonoscopy and sigmoidoscopy (or endoscopy) in Switzerland (6.4% in 2007 and 15.0% in 2012),^59^^,^^60^ Canada (37.2% in 2012),^57^ and Korea (18.5% in 2013).^49^

Regarding utilization of sigmoidoscopy only, a study from Singapore provided estimates, both overall and by sex indicating low 5-year utilization among eligible adults (data from 2007/2008: 10.8%), with slightly higher utilization observed in men.^62^

The overall utilization pattern of endoscopy in our extracted data revealed a higher rate in men and among older groups in most countries^45^^,^^48^^,^^51^^,^^53^^,^^54^^,^^57^^,^^61^^,^^62^ (Table 5, Table S7).

### Combined use of fecal tests, flexible sigmoidoscopy and/or colonoscopy in other countries

Fifteen studies from Canada, Singapore, Switzerland, Korea, Saudi Arabia, and Europe reported utilization of fecal tests, flexible sigmoidoscopy and/or colonoscopy (combined).^8^^,^^10^^,^^45^, ^46^, ^47^^,^^49^^,^^50^^,^^55^^,^^57^, ^58^, ^59^, ^60^, ^61^, ^62^, ^63^ Screening within recommended guidelines for any test in Canada increased from 30.1% in 2003^45^ to 55.2% in 2012,^57^ aligning respectively with comparable estimate reported in Korea [55.6% (2013)].^50^ In Switzerland, the overall utilization rate in 2017 (48.1%)^61^ was comparable to some other European countries based on EHIS 2013–2015 data (e.g. Denmark: 47.0%).^8^ Among European nations, Germany (70.9%) and Denmark (74.9%) exhibited the highest utilization of either fecal tests or colonoscopy among people aged 50–74 respectively, in 2013–2015 and 2018–2020, while Romania (6.3%) and Bulgaria (6.3%) reported the lowest utilization rates, respectively, in these years.^8^^,^^10^ In Saudi Arabia, CRC screening use was only 5.6% in 2007.^63^ Estimates from Korea demonstrated a substantial increase from 19.9% in 2004 to 64.4% in 2020^45^^,^^46^^,^^50^ (Table 6, Table S8).

**Table 6:** Estimates of use of any CRC Screening test in other countries.^a^

First author (Yr)	Study characteristics					CRC test utilization			
	Country	Data source	Latest year	Sample size	Age	Time frame	Overall	Men	Women
Cardoso^d^ (2020)^8^	30 European countries	EHIS	2015	125,375	50–74	FOBT/FIT: 2 yrs, Col. 10 yrs.	By country: see Table S8	NR	
Ola^d^ (2024)^10^	29 European countries	EHIS	2020	129,750	50–74	FOBT/FIT: 2 yrs, Col. 10 yrs.	By country: see Table S8	NA	
Sewitch (2007)^55^	Canada	CCHS	2003	17,498	50+	FOBT: 2 yrs, Endo: 10 yrs	30.1%	NR	
						Ever	41.7%		
Singh^d^ (2015)^57^	Canada	CCHS	2012	NR	50–74	Either or both FOBT (1 yr) and FS/Col.	55.2%	54.9%	55.5%
Simkin (2019)^58^	Canada	CCHS	2014	22,523	50–74	Either/both FOBT: 2 yrs and FS/Col: 10 yrs	52.0%	NR	
Choi^d^ (2010)^45^	Korea	KNCSS	2008	922	50–79	Col: 10 yrs, DCBE: 5 yrs; FOBT: 1 yr	36.6%	NR	
Park^d^ (2012)^46^	Korea	KNCSS	2011	21,865	50+	Col: 10 yrs, DCBE: 5 yrs; FOBT: 1 yr	35.3%	NR	
						Ever	56.1%		
Suh^d^ (2016)^47^	Korea	KNCSS	2013	30,105	50+	Col: 10 yrs, FOBT: 1 yr; DCBE: 5 yrs	55.6%	56.3%	54.9%
						Ever	70.3%	NR	
Choi^d^ (2018)^49^	Korea	KNCSS	2013	2154	50+	FOBT: 1 yr, Col: 10 yrs.	67.0%	49.6%	50.4%
Park^d^ (2022)^50^	Korea	KNCSS^c^	2020	29,040	50–74	FIT: 1 yr, DCBE: 5 yrs, or Col: 10 yrs	64.4%	67.3%	61.6%
Khoja^d^ (2018)^63^	Saudi Arabia	SNSEH	2006/7	2946	60+	FOBT: 1 yr; Col: 5 yrs.	5.6%	5.9%^b^	5.4%^b^
Wong (2013)^62^	Singapore	HH survey	2007/8	1743	50+	FOBT- 1 yr, FS: 5 yrs; Col.- 10 yrs	26.7%	28.7%	25.4%
Spaeth (2013)^59^	Switzerland	SHIS	2007	NR	50+	FOBT- 1 yr, Endo: 5 yrs	13.0%	NR	
Fedewa^d^ (2015)^60^	Switzerland	SHIS	2012	7224	50–75	FOBT: 2 yrs, Endo: 10 yrs	22.2%	NR	
Schneider^d^ (2022)^61^	Switzerland	SHIS	2017	8038	50–75	Any test	48.1%	48.2%	47.9%

Abbreviations: CCHS, Canadian Community Health Survey; HH survey, Household survey; KNCSS, Korean National Cancer Screening Survey; SHIS, Swiss Health Interview Survey; SNSEH, Saudi National Survey for Elderly Health; EHIS, European Health Interview Survey; FOBT, fecal occult blood test; FIT, fecal immunochemical test; Endo, endoscopy; Col., colonoscopy; FS, flexible sigmoidoscopy; DCBE, double-contrast barium enema; NR, not reported; yr(s), year(s).

a Table ordered first according to country (in alphabetical order), then the year of data collection.

b Data calculated from the available information in the article.

c Up-to-date with CRC screening was defined as FIT within 1 year, or DCBE within 5 years, or colonoscopy within 10 years in 2005–2018. This was changed to FIT within 1 year or colonoscopy within 10 years in 2019–2020.^50^

d Other information (e.g. estimates for other years reported in the study, or estimates by age or sex) are provided in Table S8).

### Description of temporal trends in CRC screening test use

**Fig. 2:**
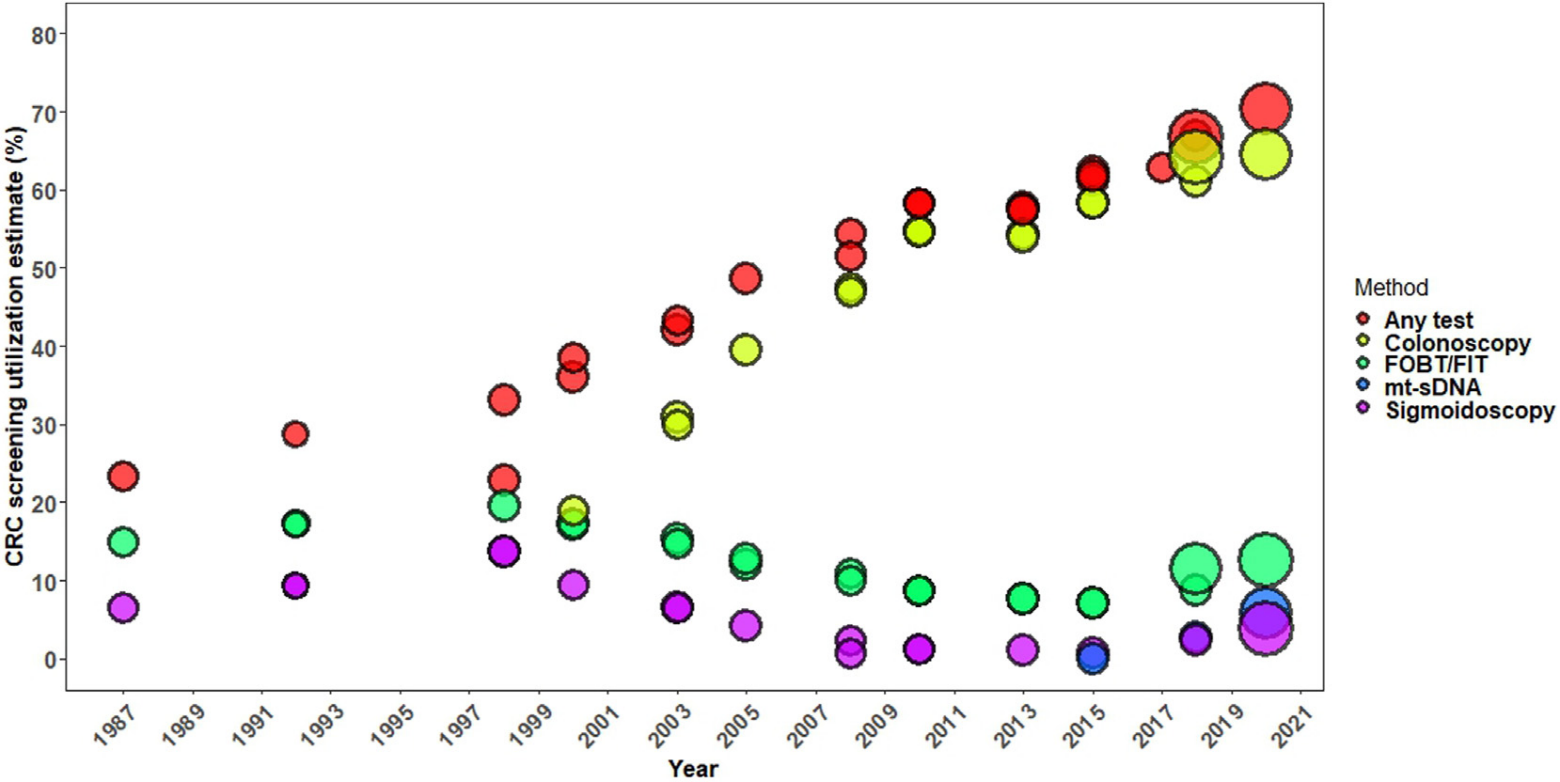
Trends in CRC screening utilization in the US, 1987–2020. Circle size represents relative sample size. Only studies that used test intervals consistent with national guidelines were included in the examination of trends; i.e fecal occult blood test/fecal immunochemical test (FOBT/FIT) within 1 year, colonoscopy within 10 years, sigmoidoscopy or computed tomographic (CT) colonography within 5 years, or multitarget stool DNA (mt-sDNA) within 3 years.

Using data from included studies from 1987,^18^, ^19^, ^20^^,^^22^^,^^24^, ^25^, ^26^^,^^28^^,^^29^^,^^32^^,^^33^^,^^35^^,^^40^^,^^41^^,^^43^ we observed a steady rise in use of any CRC screening test from approximately 24% in 1987 to 70% in 2020 in the US This increase was predominantly driven by a steep increase in colonoscopy utilization, from nearly 20% in 2000 to 64.5% in 2020.^29^^,^^35^ In contrast, following an initial rise up to 1998, both fecal tests and sigmoidoscopy utilization continuously declined from approximately 20% (fecal tests) and 13.6% (sigmoidoscopy) in 1998 to as low as 7.1% and less than 1%, respectively, in 2015.^19^^,^^20^^,^^32^ However, there has been a slight increase in the utilization of these tests since 2016, along with the newly-introduced mt-sDNA (5.8% in 2020)^35^ (Fig. 2).

**Fig. 3:**
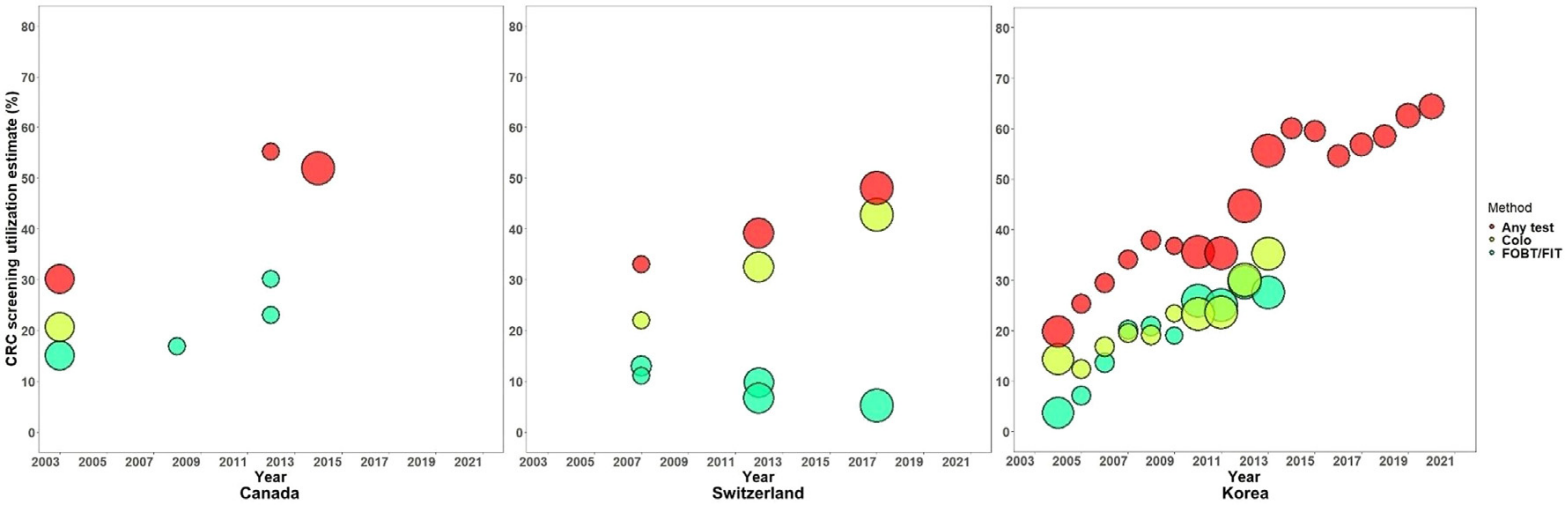
Trends in CRC screening utilization in other countries (Canada, Switzerland, and Korea, 2003–2020). Countries with estimates for at least three time points and with test intervals consistent with respective national guidelines were included in the examination of trends. Circle size represents relative sample size. FIT, fecal immunochemical test. Until 2009, up-to-date screening in Korea involved the utilization of either colonoscopy within 5 years, double-contrast barium enema (DCBE) within 5 years, or fecal occult blood test (FOBT) within a year. Subsequently, the interval for colonoscopy was extended to 10 years, and DCBE was excluded from the recommendations starting in 2018.^47^^,^^54^

**Fig. 4:**
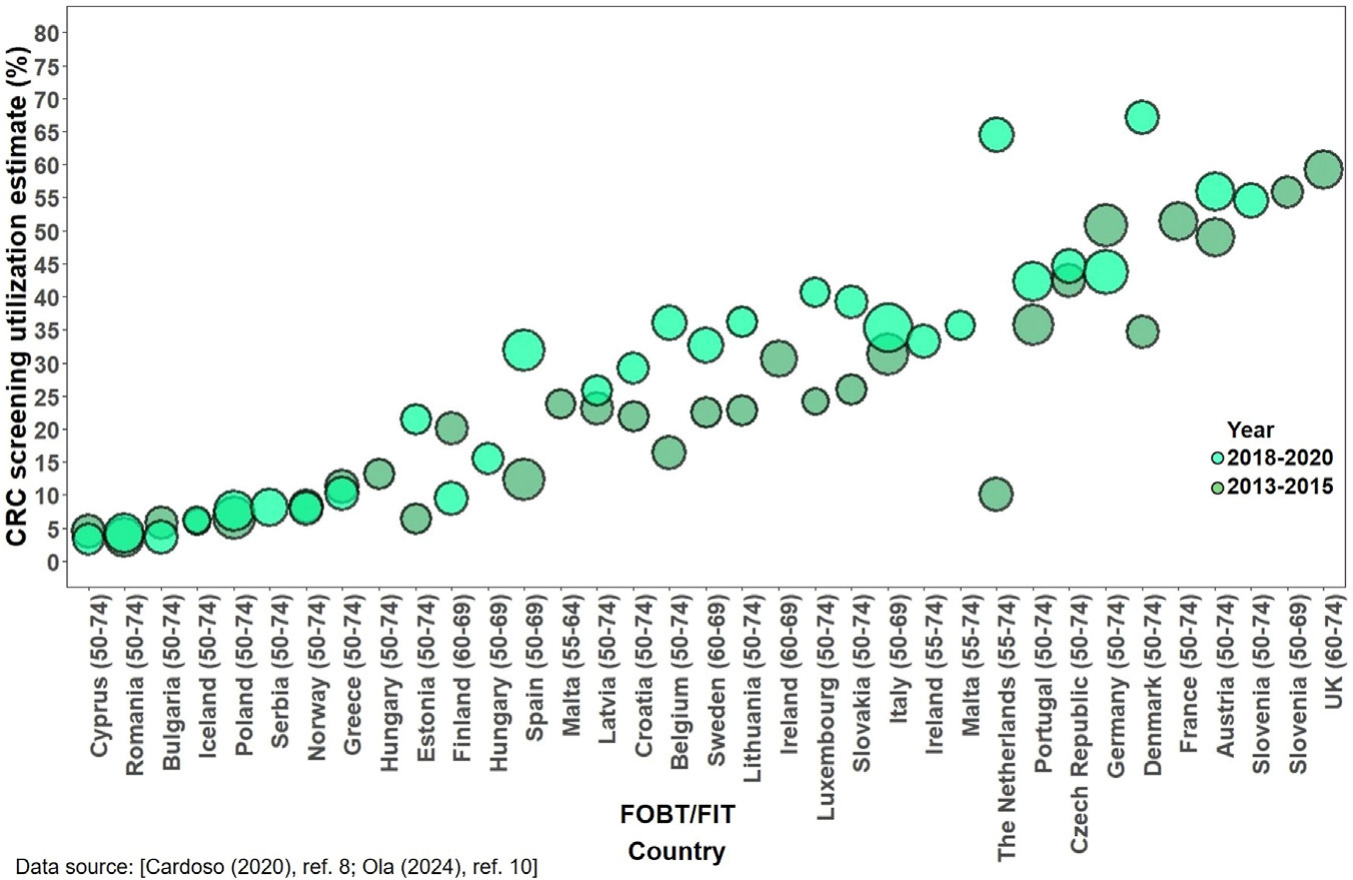
Fecal tests (FOBT/FIT) utilization in Europe in 2013–2015 and 2018–2020.

**Fig. 5:**
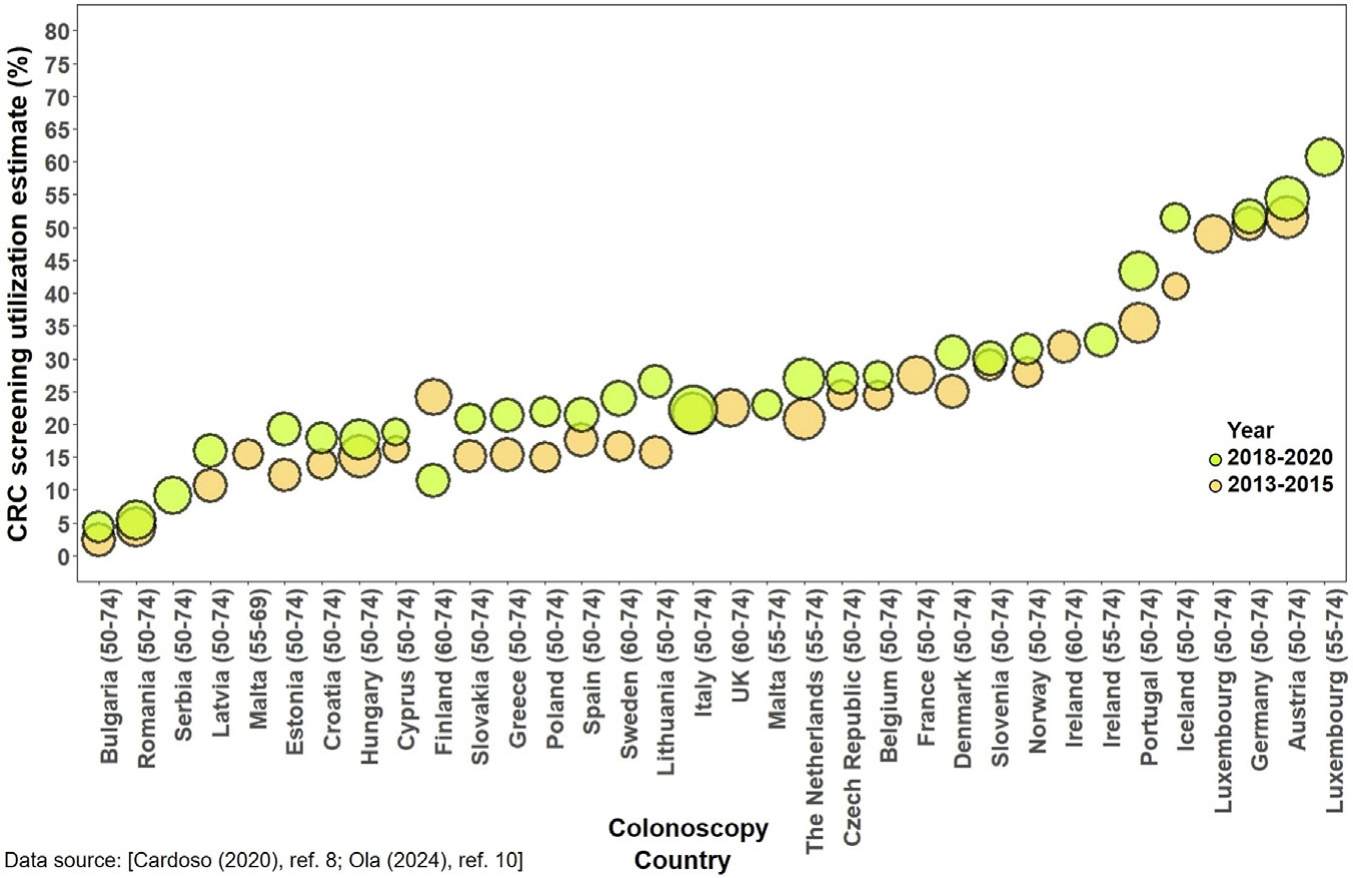
Colonoscopy utilization in Europe in 2013–2015 and 2018–2020.

**Fig. 6:**
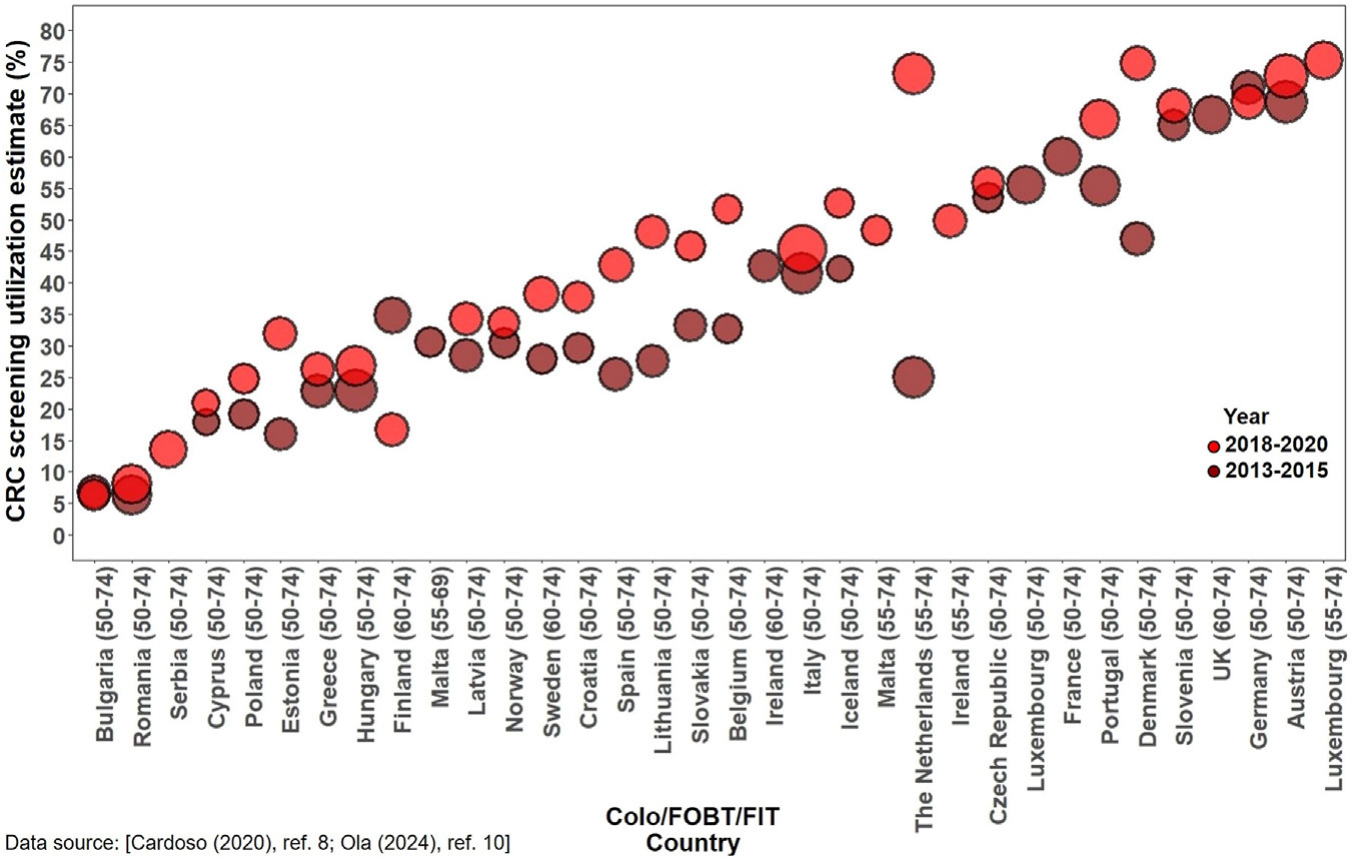
Utilization of either test in Europe in 2013–2015 and 2018–2020.

The utilization of any screening test continuously increased over time in Korea,^47^^,^^50^ Switzerland,^60^^,^^61^ Canada,^55^, ^56^, ^57^^,^^58^ and most of Europe.^8^^,^^10^ Despite slight downturns in 2009–2011 and 2016–2017, the use of any screening tests consistently increased in Korea, driven almost equally by both fecal tests and colonoscopy, mirroring the pattern observed in Canada Conversely, in Switzerland, the prevalence of CRC screening is predominantly propelled by colonoscopy, while the use of fecal tests steadily declined (Fig. 3, Fig. 4, Fig. 5, Fig. 6).

## Discussion

This review summarizes national estimates of CRC screening test utilization among eligible age groups across different countries. The overall usage of CRC screening tests showed an upward trend over time in the majority of the countries examined in this review, accompanied by considerable variations in the extent of utilization for each test. Nevertheless, the estimates remained below desired levels of utilization, as most national targets are unmet to date.

The upward trend observed in the US was driven by the rapid rise in colonoscopy utilization since the early 2000s.^4^^,^^11^^,^^22^ However, recent studies indicate a new surge in the adoption of gFOBT/FIT in the US, as well as the increasing use of mt-sDNA.^32^^,^^33^^,^^66^ The surge in colonoscopy utilization is likely strongly linked to its recommendation by US physicians.^67^ Indeed, despite the challenges associated with colonoscopy, including its high cost, need for rigorous bowel preparation, and potential complications due to its invasive nature,^4^ a significant proportion of US primary care physicians reported a considerable uptick in recommending colonoscopy compared to fecal tests, while the volume of recommended sigmoidoscopies decreased substantially.^67^ This can be partly attributed to the perception of colonoscopy as the gold standard. However, it is also viewed as more readily available, and concerns about potential legal repercussions if not offered to patients have contributed to the significant increase in its recommendation.^67^

Although colonoscopy has made a significant contribution to accelerating population CRC screening use in the US, there is concern about its potential to exacerbate disparities in healthcare access. As colonoscopy continues to be the predominant screening method, disparities in CRC screening uptake persist along ethnic and racial lines, socioeconomic status, and among the uninsured and underinsured populations.^27^^,^^35^^,^^42^^,^^68^^,^^69^

Except for mt-sDNA, which is only approved in the US, fecal tests have gained widespread adoption in various parts of the world as the primary screening method, with colonoscopy primarily reserved for follow-up tests following a positive fecal test.^4^ In Europe, the EU Commission's Independent Expert Report on Cancer Screening recommended FIT as the triage test in organized CRC screening programs.^70^ While recognizing the superior performance of endoscopic screenings, FIT was considered more acceptable, cost-effective, and required less highly skilled manpower.^70^ These factors contributed to the growing utilization of fecal tests beyond the US. They are equally relevant to resource-constrained regions in Asia, South America, and sub-Saharan Africa, offering an opportunity to enhance screening uptake and alleviate technical and logistical barriers to screening. In the US, the cumulative impact of physicians' favorable attitudes towards colonoscopy,^67^ and its unique advantages as the sole single-step test encompassing screening, diagnostic, and therapeutic capabilities,^13^ might have played a role in its prioritization over fecal tests.

Similarly, in contrast to organized screening systems where a two-step approach involving fecal tests could prove effective, the predominantly opportunistic nature of CRC screening in the US makes fecal tests less favorable as a first-line method. This, in part, has influenced the country's CRC screening guidelines.^14^ Following the US Centers for Medicare and Medicaid Services policy that necessitated co-payment for follow-up colonoscopy after a positive fecal test, in contrast to direct screening colonoscopy, the incentive for fecal test utilization was effectively diminished.^71^

Various factors may account for the gradual decline in the utilization of FS for CRC screening. The requirement for a follow-up colonoscopy when polyps are detected during screening FS renders it relatively cost-inefficient.^4^ Additionally, its long-term efficacy in reducing CRC incidence and mortality among women is uncertain.^72^ Considering the affordability and procedural simplicity of gFOBT/FIT compared to FS, this might also affect adherence to FS among eligible populations. Moreover, the clinical guidelines from the American College of Gastroenterology recommend FS primarily for individuals with incomplete colonoscopy or those who are unfit or unwilling to undergo colonoscopy or FIT.^14^

While there was notable variability in sex-specific screening utilization over time, the overall age-specific pattern consistently revealed low utilization among younger age groups across all countries and screening methods. Given the anticipated increase in CRC incidence and mortality in the coming decades,^73^ the absence of targeted strategies to promote screening uptake among younger, eligible age groups within many existing screening guidelines and programs could exacerbate this detrimental trend.

In the studies reviewed, we observed that a majority of the existing data and population surveys inadequately distinguish between screening and diagnostic tests for CRC. There has been an argument suggesting that differentiating between screening and non-screening CRC tests is inconsequential, as repeat screening tests are deemed unnecessary even when initially conducted for non-screening purposes.^33^^,^^57^ Nonetheless, segregating the tests facilitates a precise assessment of screening program effectiveness, preventive health education, and the identification and potential resolution of barriers and disparities in access and utilization of these tests.^20^

Despite the proven reliability of self-reported surveys,^74^ achieving complete discrimination between screening and non-screening tests remains challenging.^75^ However, incorporating comprehensive supporting information in the surveys and encouraging interviewers, where applicable, to diligently explain the distinctions can be beneficial. Additionally, the creation of distinct questions for screening and non-screening CRC tests in surveys may prompt respondents to think critically about the accuracy of the information they provide.

Due to the paucity of studies^7^ and our emphasis on nationally representative reports, this review did not include estimates from countries in Africa, South America, and much of Asia. This may potentially reflect the limited availability of CRC screening tests, the absence of population-based screening programs, or the lack of research-centric population health surveys and databases in these regions. Nevertheless, the cost-effectiveness of screening colonoscopy and FIT has been established in some parts of Asia and Africa,^76^^,^^77^ and feasibility studies have been suggested to explore the implementation of FIT-based screening in Africa.^78^

A recent review identified a lack of infrastructure and trained personnel for endoscopy, along with insufficient patient education about CRC screening, as barriers to screening in Africa.^78^ Addressing these potential barriers is crucial, and there is a need to integrate proven screening strategies tailored to local resources and technical and logistical peculiarities.

In a large population like China, most CRC screening programs are predominantly conducted at provincial and municipal levels, with two central and four regional-level programs initiated as of 2020.^79^ However, most research has focused on evaluating program-specific metrics rather than the overall population-level screening utilization rates. A recent review showed that the national average screening coverage among the eligible age group (40–74 years) across these existing programs—including organized, opportunistic, and physical examination pathways—remains approximately 3%.^79^

Progressively, as many countries strive to achieve diverse population CRC screening utilization targets, the emphasis should be on enhancing accessibility, affordability, eliminating disparities in access, and adopting resource-effective strategies. The implementation of organized CRC screening programs has proven effective in enhancing screening uptake, for example in Europe, particularly when implemented on a national scale.^8^^,^^10^

Also, the implementation of organized screening guidelines with FIT could empower physicians in their messaging and recommendations. It has been demonstrated to rapidly increase CRC screening utilization, particularly in regions previously operating opportunistic programs.^80^ The recent American College of Gastroenterology guidelines recommending a FIT-based organized system could further accelerate CRC screening utilization.^14^ Moreover, the concurrent implementation of the new US Centers for Medicare and Medicaid Services cost coverage for follow-on colonoscopy may offer synergistic support, contributing to the reduction of disparities in CRC screening.^71^

Furthermore, the availability of multiple screening alternatives could attract individuals with diverse screening preferences.^14^ Combining various strategies to mobilize eligible individuals is likely to enhance test utilization, even within organized screening systems.^14^^,^^81^

A key strength of this study is its inclusion of studies exclusively utilizing nationally representative data. The analysis of time trends in CRC screening test utilization, especially in the US, offers a comprehensive overview of the progress achieved over the past three decades, providing an opportunity to understand how various policies and guidelines have impacted screening utilization.

A limitation of the study is that majority of eligible studies were identified in the US, largely owing to the consistent availability of population-based data like NHIS over several decades. In contrast, eligible studies from other regions are limited or sometimes absent, making it challenging to form a comprehensive understanding of screening utilization over the long term. Similarly, we cannot rule out potential biases that might have been introduced due to non-inclusion of studies which utilized regional/provincial-level data or data from large population subgroups.

Additionally, since the included studies predominantly relied on self-reported data, the risk of recall and reporting biases, with the potential for both overestimation and underestimation of screening test use, cannot be ruled out.

Overall, CRC screening tests remain underutilized in many countries, notwithstanding the rising colonoscopy-driven trends in the US and the increasing adoption of triage fecal tests in Europe and a few other countries. Efforts to reduce CRC incidence and mortality rely on wide coverage and utilization of proven screening tests by all eligible individuals. Hence, without discarding other screening options, including sigmoidoscopy, the implementation of organized, FIT-based programs could be a cost-efficient strategy to rapidly increase screening uptake, even in resource-limited settings.

Distinguishing between screening and non-screening tests in surveys is useful. Where resources permit, regular population-based surveys are encouraged to facilitate prompt, policy-relevant decision-making for addressing screening uptake and various barriers to screening utilization.

## Contributors

Conceptualization of topic: I.O., R.C., and H.B.; Research methodology: I.O., R.C., M.H., and H.B. Data curation: I.O. and R.C.; analysis, software, and investigation: I.O.; resources: H.B. Writing—Original draft preparation: I.O.; Writing—Co-authors review and editing: I.O., R.C., M.H., and H.B.; Supervision: H.B. All authors have read and approved the submitted version. All the authors verified the underlying data used in the study.

## Data sharing statement

The data used in this study is available as published data in the various included articles. Data inventories are also provided as supplementary materials.

## Declaration of interests

The authors declare no conflicts of interest in the conduct of this study.
