# Supplementary material for: Utilization of colorectal cancer screening tests: a systematic review and time trend analysis of nationally representative data
Source: eClinicalMedicine. 2024 Aug 21;75:102783. doi: 10.1016/j.eclinm.2024.102783 (PMC11388351; doi:10.1016/j.eclinm.2024.102783)
Supplement: Supplementary Appendix 1 [file mmc1.docx]

**Utilization of Colorectal Cancer Screening Tests:**

**A Systematic Review and Time Trend Analysis of Nationally Representative Data**

**Idris Ola ^1, 2^ Rafael Cardoso^1^ Michael Hoffmeister ^1^ Hermann Brenner ^1, 3, 4^**

## **SUPPLEMENTARY APPENDIX 1**

**Table of contents**

Page no.

1. Title and table of contents 1
2. Table S1. Search strategy and outputs in electronic databases 2
3. Table S2. EPHPP quality assessment and ratings of included studies. 3
4. Table S3. Estimates of recent and lifetime use of fecal test from the United States. 4-5
5. Table S4. Estimates of recent and lifetime use of lower GI endoscopy in the United States. 6-7
6. Table S5: Estimates of use of any CRC Screening test in the United States. 8-9
7. Table S6. Estimates of recent and lifetime use of fecal test in other countries. 10-12
8. Table S7. Estimates of recent and lifetime use of lower gastrointestinal endoscopy in other countries. 13-15
9. Table S8. Estimates of use of any CRC Screening test in other countries. 16-18

**Table S1. Search strategy and outputs in electronic databases.**

| **No.** | **Query** | **Output** |
| --- | --- | --- |
| **PubMed/Medline** | | |
| **1** | ((((colorectal) OR (bowel)) OR ("colon"[[MeSH Terms])) OR (colon[Text Word])) OR (rectal) | 954,706 |
| **2** | ((((cancer[Text Word]) OR ("neoplasms"[MeSH Terms])) OR (neoplasm*[Text Word])) OR ("carcinoma"[MeSH Terms])) OR (carcinoma[Text Word]) | 4,795,057 |
| **3** | (((((((((((((("fecal test*") OR ("faecal test*")) OR ("occult blood"[MeSH Terms])) OR (fecal occult blood test[Text Word])) OR ("fecal immunochemical test*")) OR ("faecal immunochemical test*")) OR ("colonoscopy"[MeSH Terms])) OR (colonoscopy[Text Word])) OR (colonoscop*)) OR ("endoscopy"[MeSH Terms])) OR (endoscopy[Text Word])) OR (endoscop*)) OR ("sigmoidoscopy"[MeSH Terms])) OR (sigmoidoscopy[Text Word])) OR (sigmoidoscop*) | 582,736 |
| **4** | (((((((("mass screening"[MeSH Terms]) OR ("early detection of cancer"[MeSH Terms])) OR (screening[Text Word])) OR (examinat*)) OR ("mass screening")) OR ("population-based screening")) OR ("community-based screening")) OR ("surveillance")) OR ("strateg*") | 3,652,018 |
| **5** | **#1 AND #2** | 406,014 |
| **6** | **#3 AND #4** | 103,955 |
| **7** | **#5 AND #6** | 24,882 |
| **8** | Filtering (Not relevant to study): Books, Documents, Meta-analyses, Reviews, Systematic reviews | 3,563 |
| **Web of Science** | | |
| **1** | ((((((TI=(colorectal)) OR AB=(colorectal)) OR AB=(bowel)) OR TI=(bowel)) OR ALL=("colon")) OR ALL=(colon)) OR ALL=(rectal) | 678,145 |
| **2** | (((((((((TI=(cancer)) OR AB=(cancer)) OR AB=("neoplasms")) OR TI=("neoplasms")) OR TI=(neoplasm*)) OR AB=(neoplasm*)) OR AB=("carcinoma")) OR TI=("carcinoma")) OR TI=(carcinoma)) OR AB=(carcinoma) | 3,095,777 |
| **3** | (((((((((((((((((((((((((((((TI=(“fecal test*” )) OR AB=(“fecal test*” )) OR AB=(“faecal test*” )) OR TI=(“faecal test*” )) OR TI=("occult blood")) OR AB=("occult blood")) OR AB=(fecal occult blood test)) OR TI=(fecal occult blood test)) OR TI=(“fecal immunochemical test*”)) OR AB=(“fecal immunochemical test*”)) OR AB=(“faecal immunochemical test*” )) OR TI=(“faecal immunochemical test*” )) OR TI=("colonoscopy")) OR AB=("colonoscopy")) OR AB=(colonoscopy)) OR TI=(colonoscopy)) OR TI=(colonoscop*)) OR AB=(colonoscop*)) OR AB=("endoscopy")) OR TI=("endoscopy")) OR TI=(endoscopy)) OR AB=(endoscopy)) OR AB=(endoscop*)) OR TI=(endoscop*)) OR TI=("sigmoidoscopy")) OR AB=("sigmoidoscopy")) OR AB=(sigmoidoscopy)) OR TI=(sigmoidoscopy)) OR TI=(sigmoidoscop*)) OR AB=(sigmoidoscop*) | 266,862 |
| **4** | (((((((((((((((((TI=("mass screening")) OR AB=("mass screening")) OR AB=("early detection of cancer")) OR TI=("early detection of cancer")) OR TI=(screening)) OR AB=(screening)) OR AB=(examinat*)) OR TI=(examinat*)) OR TI=("mass screening" )) OR AB=("mass screening" )) OR AB=("population-based screening")) OR TI=("population-based screening")) OR TI=("community-based screening" )) OR AB=("community-based screening" )) OR AB=("surveillance")) OR TI=("surveillance")) OR TI=(“strateg*”)) OR AB=(“strateg*”) | 4,233,283 |
| **5** | **#1 AND #2** | 334,606 |
| **6** | **#3 AND #4** | 53,395 |
| **7** | **#5 AND #6** | 13,688 |
| **8** | Filtering: Limited to relevant micro- and then meso-level citation topics (liver and colon cancer). | 5,065 |
| **Embase** | | |
| **1** | (colorectal)/br OR ((bowel):ab,ti) OR (("colon"):ab,ti) OR ((colon):ab,ti) OR ((rectal):ab,ti) | 977,571 |
| **2** | (cancer)/br OR (("neoplasms"):ab,ti) OR ((neoplasm*):ab,ti) OR (("carcinoma"):ab,ti) OR ((carcinoma):ab,ti) | 6,660,122 |
| **3** | (“fecal test*”)/br OR ((“faecal test*”):ab,ti) OR (("occult blood"):ab,ti) OR (('fecal occult blood test'):ab,ti) OR ((“fecal immunochemical test*”):ab,ti) OR ((“faecal immunochemical test*”):ab,ti) OR (("colonoscopy"):ab,ti) OR ((colonoscopy):ab,ti) OR ((colonoscop*):ab,ti) OR (("endoscopy"):ab,ti) OR ((endoscopy):ab,ti) OR ((endoscop*):ab,ti) OR (("sigmoidoscopy"):ab,ti) OR ((sigmoidoscopy):ab,ti) OR ((sigmoidoscop*):ab,ti) | 479,452 |
| **4** | ("mass screening")/br OR (("early detection of cancer"):ab,ti) OR ((screening):ab,ti) OR ((examinat*):ab,ti) OR (("mass screening"):ab,ti) OR (("population-based screening"):ab,ti) OR (("community-based screening"):ab,ti) OR (("surveillance"):ab,ti) OR ((“strateg*”):ab,ti) | 4,615,249 |
| **5** | **#1 AND #2** | 573,374 |
| **6** | **#3 AND #4** | 122,985 |
| **7** | **#5 AND #6** | 36,994 |
| **8** | Filtering: Duplicated sources from Embase and Medline, Medline, age <65, study types, publication types, conference abstracts, and disease types not relevant to topic. | 537 |

**Table S2. Effective Public Health Practice Project (EPHPP) quality assessment and ratings of included studies.^#^**

| **First author (Yr)** | **Study Design** | **Selection bias** | **Confounders** | **Blinding (If Applicable)** | **Data Collection method** | **Withdrawals and dropouts** | **Global Rating** |
| --- | --- | --- | --- | --- | --- | --- | --- |
| Anderson (1995) | 3 | 1 | 1 | NA | 1 | 3 | 3 |
| Breen (2001) | 3 | 1 | 1 | NA | 1 | 1 | 2 |
| Nadel (2002) | 3 | 1 | 1 | NA | 1 | 1 | 2 |
| Swan (2003) | 3 | 1 | 1 | NA | 1 | 2 | 2 |
| Seef (2004) | 3 | 1 | 1 | NA | 1 | 1 | 2 |
| Subramanian (2005) | 3 | 1 | 1 | NA | 1 | 1 | 2 |
| Liang (2006) | 3 | 1 | 1 | NA | 1 | 1 | 2 |
| Meissner (2006) | 3 | 1 | 1 | NA | 1 | 3 | 3 |
| Shapiro (2008) | 3 | 1 | 1 | NA | 1 | 2 | 2 |
| Doubeni (2010) | 3 | 1 | 1 | NA | 1 | 3 | 3 |
| Swan (2010) | 3 | 1 | 1 | NA | 1 | 3 | 3 |
| Klabunde (2011) | 3 | 1 | 1 | NA | 1 | 2 | 2 |
| Shapiro (2012) | 3 | 1 | 1 | NA | 1 | 2 | 2 |
| Cole (2012) | 3 | 1 | 1 | NA | 1 | 3 | 3 |
| de Moor (2018) | 3 | 1 | 1 | NA | 1 | 3 | 3 |
| Shapiro (2021) | 3 | 1 | 1 | NA | 1 | 3 | 3 |
| Zhu (2021) | 3 | 2 | 2 | NA | 1 | 3 | 3 |
| He (2023) | 3 | 1 | 1 | NA | 1 | 2 | 2 |
| Star (2024) | 3 | 1 | 1 | NA | 1 | 3 | 3 |
| Chao (2004) | 2 | 1 | 1 | NA | 1 | 1 | 2 |
| Sanford (2019) | 3 | 2 | 1 | NA | 1 | 3 | 3 |
| Hong (2021) | 3 | 1 | 1 | NA | 1 | 1 | 2 |
| Sabatino (2015) | 3 | 1 | 1 | NA | 1 | 2 | 2 |
| White (2017) | 3 | 1 | 1 | NA | 1 | 3 | 3 |
| Sokale (2022) | 3 | 1 | 1 | NA | 1 | 3 | 3 |
| Santiago-Rodríguez (2023) | 3 | 1 | 1 | NA | 1 | 2 | 2 |
| Castañeda-Avila (2024) | 3 | 1 | 1 | NA | 1 | 3 | 3 |
| Cardoso (2020) | 3 | 1 | 1 | NA | 1 | 2 | 2 |
| Ola (2024) | 3 | 1 | 1 | NA | 1 | 2 | 2 |
| Wahidie (2023) | 3 | 1 | 1 | NA | 1 | 3 | 3 |
| Sewitch (2007) | 3 | 1 | 1 | NA | 1 | 2 | 2 |
| Major (2015) | 3 | 1 | 1 | NA | 1 | 3 | 3 |
| Singh (2015) | 3 | 1 | 1 | NA | 1 | 3 | 3 |
| Sieverding (2010) | 3 | 1 | 1 | NA | 1 | 3 | 3 |
| Wahidie (2022) | 3 | 1 | 1 | NA | 1 | 3 | 3 |
| Choi (2010) | 3 | 1 | 1 | NA | 1 | 3 | 3 |
| Park (2012) | 3 | 1 | 1 | NA | 1 | 3 | 3 |
| Suh (2016) | 3 | 1 | 1 | NA | 1 | 3 | 3 |
| Bui (2017) | 3 | 1 | 1 | NA | 1 | 3 | 3 |
| Choi (2018) | 3 | 1 | 1 | NA | 1 | 2 | 2 |
| Khoja (2018) | 3 | 1 | 1 | NA | 1 | 3 | 3 |
| Wong (2013) | 3 | 1 | 1 | NA | 1 | 1 | 2 |
| Portero de la Cruz (2023) | 3 | 1 | 1 | NA | 1 | 3 | 3 |
| Spaeth (2013) | 3 | 1 | 1 | NA | 1 | 2 | 2 |
| Fedewa (2015) | 3 | 1 | 1 | NA | 1 | 3 | 3 |
| Schneider (2022) | 3 | 1 | 1 | NA | 1 | 3 | 3 |
| Starker (2017) | 3 | 1 | 1 | NA | 1 | 3 | 3 |
| Hornschuch (2022) | 3 | 1 | 2 | NA | 2 | 3 | 3 |
| Simkin (2019) | 3 | 1 | 1 | NA | 1 | 2 | 2 |
| Park (2022) | 3 | 1 | 1 | NA | 1 | 3 | 3 |

**^#^** For each domain, studies are scored 1= Strong; 2= Moderate; 3= Weak. The Global rating aggregate the scores from each domain and assign scores as follows: 1 = Strong (no weak ratings); 2 = Moderate (one weak rating); 3 = Weak (two or more weak ratings).^17^ NA, not applicable; yr, year(s).

**Table S3. Prevalence estimates of recent and lifetime use of fecal test (gFOBT or FIT, or mt-sDNA) from the United States.^a^**

| **First author (yr)** | **Data Source** | **Year(s)** | **Sample Size** | **Time frame** | **Overall** | **By age** | | | | **Men** | **Women** |
| --- | --- | --- | --- | --- | --- | --- | --- | --- | --- | --- | --- |
| Anderson (1995)^18^ | NHIS | 1987-1992 | 1987: 8240  1992: 4428 | 3 yrs | 1987: 21.7%  1992: 26.3% | **1987:** 50-64: 21.1%;  65-74: 24.7%**;** 75+: 18.4% | | **1992:** 50-64: 25.5%;  65-74: 28.1%; 75+: 25.6% | | **1987:** 20.3%  **1992:** 25.4% | **1987:** 22.8%  **1992:**26.9% |
|  |  |  |  | 1 yr | 1987: 14.9%  1992: 17.3% | NR | | | | NR | |
| Breen (2001)^19^ | NHIS | 1987-1998 | 1987: 7803  1992: 4368  1998:11925 | 2 yrs | 1987: 19.9%^b^  1992: 24.4%^b^  1998: 27.1%^b^ | 1987  50-64: 19.1%^b^  >=65: 20.7%^b^ | 1992  50-64: 24.0%^b^  >=65: 24.9%^b^ | | 1998  50-64: 25.4%^b^  >=65: 29.2%^b^ | 1987:18.2%  1992: 23.8%  1998: 28.5% | 1987: 20.9%  1992: 24.8%  1998: 26.1% |
| Nadel (2002)^20^ | NHIS | 1992-1998 | 1992: 4292  1998:12128 | 1 yr | 1992: 17.2% (15.8-18.6)  1998: 19.5% (18.7-20.3) | **NR** | | | | **NR** | |
| Swan (2003)^21^ | NHIS | 2000 | 11622 | 1 yr | 1987: 20%^b^; 1992: 23.4%^b^  1998: 26.0%^b^; 2000: 25%^b^ | NR | | | | **2000:** 24.0% | 26.0% |
| Seef (2004)^22^ | NHIS | 2000 | 11480 | 1 yr | 17.1% (16.3-17.9) | **50-59:** 14.7%; **60-69:** 17.8%; **70-79:** 22.6%; **>=80**: 14.1% | | | | 16.8% | 17.5% |
| Liang (2006)^24^ | NHIS | 2000-2003 | 2000:11574  2003:11779 | Ever | 2000: 15.1%^b^; 2003: 13.6%^b^ | NR | | | | NR | |
|  |  |  |  | 1 yr | 2000: 9.3%^b^; 2003: 6.6%^b^ | NR | | | | NR | |
| Meissner (2006)^25^ | NHIS | 2003 | 11548 | 1 yr | 15.6% | **50-64**: 13.9%^b^; **>=65**: 18.2%^b^ | | | | 16.1% | 15.3% |
| Shapiro (2008)^26^ | NHIS | 2005 | 11,918 | 1 yr | 12.0% (11.3-12.7) | **50-59**: 9.3%; **60-69:** 14.4%; **70-79:** 14.4%; **z80**: 11.5% | | | | 12.6% | 11.6% |
| Doubeni (2010)^27^ | MCBS | 2000-2005 | 2000: 8,330  2003: 7,889  2005: 7,614 | 2 yrs | 2000: 14.9%  2003: 11.7%  2005: 10.4% | NR | | | | NR | |
| Swan (2010)^28^ | NHIS | 2005 | 11890 | 1 yr | 12.0%^b^ | **50-64:** 10.8%^b^; **>=65:** 13.7%^b^ | | | | 12.3% | 11.8% |
| Klabunde (2011)^29^ | NHIS | 2000-2008 | 2000: 10041  2003: 9928  2005: 10733  2008: 7776 | 1 yr | 2000: 17.4% (16.4-18.3)  2003: 14.8% (13.8-15.9)  2005: 12.7% (11.8-13.6)  2008: 10.9% (10.0-12.0) | NR | | | | NR | |
| Shapiro (2012)^30^ | NHIS | 2010 | 8,952 | 1 yr | 8.8% | **50-59**: 7.0%; **60-69**: 10.5%; **70-75**: 10.8% | | | | 8.9% | 8.7% |
| Cole (2012)^31^ | BRFSS | 2005 | 466,175 | FOBT: 1 yr | 1998: 8%^b;^ 2000: 9.3%^b^  2002: 10.2%^b;^ 2003: 9.5%^b^  2005: 8.8%^b^ | NR | | | | NR | |
| de Moor (2018)^32^ | NHIS | 2008-2015 | 2015: 12,541 | 1 yr | 2008: 10.0%; 2010: 8.8%  2013: 7.7%; 2015: 7.1% | 2015: 50-64: 5.9%; 65-75: 9.6% | | | | 2015: 7.5% | 6.8% |
| Shapiro (2021)^33^ | NHIS | 2018 | 10595 | gFOBT/FIT- 1 yr | 8.8% | **50-54:** 8.8%; **55-59:** 10.2%  **60-64:** 10.6%; **65-69:** 13.9%; **70-75**: 13.8% | | | | 11.7% | 10.8% |
|  |  |  |  | mt-sDNA: 3 yrs | 2.7% |  |  |  |  |  |  |
| Zhu (2021)^34^ | Household survey **^c^** | 2019 | 1595 | gFOBT: Ever | 47.5% | **45-54:** 35.7%; **55-64:** 41.6%; **65-75:** 61.4% | | | | 43.8% | 50.9% |
|  |  |  |  | mt-sDNA: Ever | 25.8% | **45-54:** 21.2%; **55-64**: 21.0%; **65-75**: 34.1% | | | | 24.1% | 27.1% |
| He (2023)^35^ | BRFSS | 2012-2020 | 2018:208,540  2020:175,698 | FOBT/FIT: 1 yr | 2018: 11.4%  2020: 12.6% | NR | | | | **2018**: 11.2%  **2020:** 12.2% | **2018:** 11.6%  **2020**: 13.0% |
|  | BRFSS | 2020 | 175,698 | mt-sDNA: 3 yrs**^c^** | 5.8% |  |  |  |  | 5.5% | 6.2% |
| Star (2024)^36^ | NHIS | 2019-2021 | NA | gFOBT/FIT: 1 yr | 2019: 1.2% (0.8-1.9)  2021: 2.4% (1.8-3.3) | NR | | | | NR | |

Abbreviations: NHIS, National Health Interview Survey; MCBS, Medicare Current Beneficiary Survey (MCBS) Access to Care data; BRFSS, Behavioral Risk Factor Surveillance System; FOBT, fecal occult blood test; gFOBT, guaiac-based fecal occult blood test; FIT, fecal immunochemical test; mt-sDNA, multi-target stool DNA test; NR, not reported; yr(s), year(s).

^a^ Table ordered first according to publication year, then the year of data collection. ^b^ Data calculated from the available information in the article. ^c^ Estimate was based on the current 2021 USPSTF recommendations, which defines up-to-date CRC screening as having had (1) a home stool blood test (FOBT or FIT) within 1 year, and/or (2) stool DNA within 3 years, and/or sigmoidoscopy within 5 years with FOBT or FIT within 3 years, and/or (3) colonoscopy within 10 years.

**Table S4. Prevalence estimates of recent and lifetime use of lower GI endoscopy (colonoscopy, sigmoidoscopy, or CT colonography) from the United States.^a^**

| **First author (yr)** | **Data Source** | **Year(s)** | **Sample Size** | **Time frame** | **Overall** | | **By Age** | | | | **Men** | **Women** |
| --- | --- | --- | --- | --- | --- | --- | --- | --- | --- | --- | --- | --- |
| Breen (2001)^19^ | NHIS | 1987-1998 | 1987: 7803  1992: 4368  1998: 11925 | FS: 3 yrs | 1987: 6.5%^b^  1992: 9.2%^b^  1998: 13.6%^b^ | | **1987**  50-64: 5.8%^b^  >=65: 7.4%^b^ | **1992**  50-64: 8.3%^b^  >=65: 10.3%^b^ | | **1998**  50-64:12.1%^b^  >=65: 15.3%^b^ | **1987:** 7.7%  **1992:** 12.2%  **1998:** 19.0% | **1987:** 5.8%  **1992:** 7.3%  **1998:** 9.8% |
| Nadel (2002)^20^ | NHIS | 1992, 1998 | 1992: 4311  1998: 12190 | Proc/FS:3 yrs | **1992:** 9.4% (8.4-10.4); **1998:** 13.6% (12.9-14.3) | | NR | | | | NR | |
|  |  |  |  | 5 yrs (1998) | 15.9% (15.1- 16.7) | |  |  |  |  |  |  |
| Swan (2003)^21^ | NHIS | 2000 | 11622 | Endo: 3 yrs. | **1987:** 13.0%; **1992:** 18.1%  **1998:** 23.9%; **2000:** 25% | | NR | | | | NR | |
| Seef (2004)^22^ | NHIS | 2000 | 11588 | Col: 10 yrs.  FS: 5 yrs. | 33.9% (32.9-35.0) | | **50-59:** 27%; **60-69**: 37.7%; **70-79**: 42.3%; **>=80:** 34.1% | | | | 37.4% | 31.1% |
|  |  |  |  | Ever | 38.1% | | NR | | | | NR | |
| Subramanian (2005)^23^ | NHIS | 2000 | 12,505 | Col: (5 yr.)  FS: (5 yr) | Col: 29.1%  FS: 15.3% | | **Col**: 50-65: 28.0%  65-80: 28.9%; >80: 37.7% | | **FS:** 50-65: 16.2%  65-80: 14.3%; >80: 15.4% | | **Col:** 31.8%  **FS:** 16.8% | **Col:** 26.5%  **FS:** 13.9% |
| Liang (2006)^24^ | NHIS | 2000 | 23,353 | Col: (10 yrs)  FS: (5 yrs) | **Col.**  2000:18.3%^b^  2003:29.1%^b^ | **Sig.**  2000:5.5%^b^  2003:4.2%^b^ | NR | | | | NR | |
|  |  |  |  | Ever | 2000:19.8%^b^  2003:29.1%^b^ | 2000:4.1%^b^  2003:3.0%^b^ |  |  |  |  |  |  |
| Meissner (2006)^25^ | NHIS | 2003 | Col: 11378  FS: 11395 | Col: 10 yrs  FS: 5 yrs | **Col:** 30.8%^b^  **FS:** 6.6%^b^ | | **Col:** **50-64:** 27.2%^b^; **>=65:** 35.8%^b^  **FS: 50-64**: 6.1%^b^**; >=65**: 7.3%^b^ | | | | **Col:** 32.2%  **FS:** 7.6% | **Col:** 29.8%.  **FS:** 5.9% |
| Shapiro (2008)^26^ | NHIS | 2005 | 12,045 | Endo: 10 yrs | 45.2% (44.0-46.4) | | **50-59**: 36.0%; **60-69:** 51.7%; **70-79:** 54.9%; **>=80:** 45.1% | | | | 46.9% | 43.9% |
| Doubeni (2010)^27^ | MCBS Access to Care data | 2000-2005 | 23,833 | Endo: 10 yrs. | **<5 years**  2000: 37.8  2003: 44.5%  2005: 48.9 | **>5 years**  2000:8%  2003: 8.8%  2005: 9% | NR | | | | NR | |
| Swan (2010)^28^ | NHIS | 2005 | 12016 | Col: 10 yrs  FS: 5 yrs | 21.5%^b^ | | **50-64:** 19.0%^b^; **>=65:** 25.0%^b^ | | | | 23.7% | 19.8% |
| Klabunde (2011)^29^ | NHIS | 2000-2008 | 2000: 10,041  2003: 9928  2005: 10733  2008: 7776 | Col: 10yrs  FS: 5 yrs | **Col.**  2000: 19.0% (18.0-20.1)  2003: 29.9% (28.6-31.2)  2005: 39.4% (38.0-40.9)  2008: 47.5% (45.9-49.0) | **Sig.**  2000: 9.4% (8.7-10.2)  2003: 6.6% (5.9-7.4)  2005: 4.1% (3.6-4.7)  2008: 2.4% (1.9-3.0) | NR | | | | NR | |
| Shapiro (2012)^30^ | NHIS | 2010 | Col: 8857 | 10 yrs | 54.6% (53.2-55.9) | | **50-59**: 46.3%; **60-69**: 61.9%; **70-75**: 64.5% | | | | 54.4% | 54.7% |
|  |  |  | FS: 8871 | 5 yrs | 1.3% (1.0-1.6) | | **50-59:** 0.9%; **60-69**: 1.6%; **70-75**: 2.0% | | | | 1.6% | 1.0% |
|  |  |  | CT Col: 8843 | Ever | 1.3% (1.0-1.7) | | **50-59:** 0.8%; **60-69**: 2.0%; **70-75:** 1.5% | | | | 1.7% | 1.0% |
| Cole (2012)^31^ | BRFSS | 1998-2005 | 466,175 | FS: 5 yrs or  Col. 10 yrs. | **1998**: 19.0%^b;^ **2000:** 21%^b^  **2002:** 23.5%^b;^ **2003:** 26%^b^  **2005:** 29%^b^ | | NA | | | | NA | |
| de Moor (2018)^32^ | NHIS | 2008-2015 | 2015: 12,541 | FS: 5 yrs  Col: 10 yrs | **Col.**  2008: 46.9%  2010: 54.8%  2013: 54.2%  2015: 58.3% | **Sig.**  2008: 0.7%  2010: 1.3%  2013: 1.0%  2015: 0.7% | **Sig.**  50-64: 0.6%  65-75: 0.9% | | | **Col:**  50-64: 53.4%  65-75: 68.5% | **Sig:** 1.0%  **Col:** 57.6% | **Sig:** 0.3%  **Col:** 59.1% |
| Hong (2021)^39^ | NHIS | 2015, 2018 | 34,768 | CT col: 5 yrs | 2015: 0.79%  2018: 1.33% | | **2015:** 45-49: 0.14%  50-64: 0.74%  65-75: 1.08%  76+: 1.22% | | **2018:** 45-49: 0.75%  50-64: 1.22%  65-75: 1.83%  76+: 1.51% | | **2015:** 0.85%  **2018:** 1.48% | **2015:** 0.74%  **2018:** 1.21% |
| Shapiro (2021)^33^ | NHIS | 2018 | 10595 | Col: 10 yrs | 61.1% (59.9-62.3) | | **50-54:** 42.0%; **55-59:** 59.3%; **60-64:** 66.5%  **65-69:** 68.9%; **70-75:** 73.6% | | | | 61.4% | 60.8% |
|  |  |  |  |  | **2010:** 54.6%; **2013:** 54.0%; **2015**: 58.3% | | NR | | | | NR | |
|  |  |  |  | FS: 5 yrs | 2.4% (2.1-2.8) | | **50-64:** 2.1%; **65-75:** 3.0% | | | | 2.7% | 2.1% |
|  |  |  |  | CT Col: 5 yrs | 1.0% (0.8-1.3) | | **50-64:** 0.9%; **65-75:** 1.2% | | | | 1.2% | 0.8% |
| Zhu (2021)^34^ | Household survey | 2019 | 1595 | Col: Ever | 72.2% | | **45-54**: 51.3%; **55-64**: 75%; **65-75**: 81.2% | | | | 72.5% | 72.6% |
| He (2023)^35^ | BRFSS | 2012-2020 | 2018: 208,540  2020: 175,698 | Col: 10 yrs | 2018: 64.1%  2020: 64.5% | | NR | | | | **2018:** 59.7%  **2020:** 63.3% | **2018:**63.2%  **2020:**65.3% |
|  | BRFSS | 2020 | 175,698 | FS: 5 yrs | 3.8% | |  |  |  |  | 4.7% | 3.2% |
|  |  |  |  | CT col: 5 yrs | 2.7% | |  |  |  |  | 3.2% | 2.3% |
| Star (2024)^36^ | NHIS | 2021 | NA | Col: 10 yr | 2019: 19.5% (17.6-21.5)  2021: 17.8% (16.0-19.8) | | NR | | | | NR | |

Abbreviations: NHIS, National Health Interview Survey; CPS II, Cancer Prevention Study (CPS) II Nutrition Cohort; BRFSS, Behavioral Risk Factor Surveillance System; MCBS, Medicare Current Beneficiary Survey (MCBS) Access to Care data; Endo, endoscopy; Col., colonoscopy; CT Col, computed tomographic colonoscopy; FS, flexible sigmoidoscopy; NR, not reported; yr(s), year(s).

^a^ Table ordered first according to publication year, then the year of data collection. ^b^ Data calculated from the available information in the article.

**Table S5:** **Prevalence estimates of use of any CRC screening test in the United States.^a^**

| **First author (yr)** | **Data Source** | **Year(s)** | **Sample Size** | **Time frame** | **Overall** | **By Age** | | **Men** | **Women** |
| --- | --- | --- | --- | --- | --- | --- | --- | --- | --- |
| Breen (2001)^19^ | NHIS | 1987-1998 | 1987: 7803 1992: 4368  1998: 11925 | FOBT- 2 yrs  FS: 3 yrs. | 1987: 23.4% ^b^  1992: 28.7% ^b^  1998: 33.0% ^b^ | 1998; 50-64: 30.7%^b^  >=65: 35.9%^b^ | | 1998: 37.1% | 30.2% |
| Nadel (2002)^20^ | NHIS | 1998 | 12072 | FOBT: 1 yr  Proct/FS: 5 yrs | 22.9% (22.0-23.8) | **50-59:** 19.3%; **60-69:** 26.4%  **70-79:** 27.9%; **80+:** 18.9% | | 27.1% | 19.6% |
| Swan (2003)^21^ | NHIS | 2000 | 11622 | FOBT: 1 yr or  Endo: 3 yrs. | 1987: 27.2%^b^  2000: 39.5%^b^ | 1987  50-64: 26.2%  >=65: 28.5% | 2000  50-64: 36.5%^b^  >=65: 43.2%^b^ | 1987: 26.2%  2000: 40.6%^b^ | 1987: 28.2%  2000: 38.9%^b^ |
| Seef (2004)^22^ | NHIS | 2000 | 11468 | FOBT+endo. | 42.5% (41.4-43.5) | **50-59:** 35.5%; **60-69:** 45.9%; **70-79**: 52.3%; **>=80:** 40.7% | | 44.5% | 41.0% |
|  |  |  |  | Ever | 54.2% | NR | | NR | |
| Subramanian (2005)^23^ | NHIS | 2000 | 12,505 | FOBT: 1 yr  FS: 5 yrs; Col. 10 yrs. | 25.1% | **50-65:** 22.6%; **65-80:** 30.2%; **>80:** 20.5% | | 26.7% | 23.8% |
| Liang (2006)^24^ | NHIS | 2000-2003 | 2000: 11574  2003: 11779 | FOBT: 1 yr  FS: 5 yrs or Col. 10 yrs. | 2000: 36%  2003: 42% | **2003: 50-64:** 39.3%^b^; **65-74:** 51.5%^b^  **>=75:** 47.3%^b^ | | **2003**: 45.0%^b^ | 42.0%^b^ |
|  |  |  |  | Ever | 2000: 53%  2003: 55% | **2003: 50-64:** 51.0%; **65-74:** 66.0%  **>=75:** 63.0% | | **2003**: 57.0% | 56.0% |
| Meissner (2006)^25^ | NHIS | 22003 | 11302 | FOBT: 1 yr.  FS: 5 yrs, Col-10yrs | 44.5%^b^ | **50-64:** 39.9%^b^  **>=65:** 51.2%^b^ | | 46.5% | 43.1% |
| Shapiro (2008)^26^ | NHIS | 2005 | 11,943 | FOBT: 1 yr  Endo: 10 yrs | 50.0% (48.8-51.2) | **50-59:** 40.4%; **60-69:** 56.5%; **70-79:** 60.2%; **>=80:** 50.3% | | 51.7% | 48.7% |
| Klabunde (2011)^29^ | NHIS | 2000-2008 | 2000: 10041 2003: 9928  2005: 10733  2008: 7776 | Up-to-date | 2000: 38.6% (37.4-39.9)  2003: 43.3% (41.9-44.7)  2005: 48.6% (47.2-50.1)  2008: 54.5% (52.9-56.2) | NR | | NR | |
| Shapiro (2012)^30^ | NHIS | 2010 | 8825 | FOBT: 1 yr,  FS: 5 yrs, Col.: 10yrs | 58.3% (57.0-59.6) | **50-59:** 50.0%; **60-69:** 65.8%; **70-75:** 68.2% | | 58.2% | 58.4% |
| Cole (2012)^31^ | BRFSS | 2005 | 466,175 | Up-to-date (2005): FOBT: 1 yr; FS: 5 yrs; Col. 10 yrs. | **1998:** 40.5%^b^; **2000:** 42.5%^b^  **2002:** 48.5%^b^; **2003:** 49.5%^b^; **2005:** 52.0%^b^ | NR | | NR | |
| Sabatino (2015)^40^ | NHIS | 2013 | 13045 | FOBT: 1 yr  FS: 5 yrs; Col: 10 yrs | 57.8% (56.6-59.0) | **50-64:** 52.8%  **65-75:** 69.4% | | 56.7% | 58.9% |
| White (2017)^41^ | NHIS | 2015 | 12650 | FOBT: 1 yr  FS: 5 yrs; Col: 10 yrs | 62.4% (61.1-63.7) | **50-64:** 57.9%  **65-75:** 71.8% | | NR | |
| de Moor (2018)^32^ | NHIS | 2008-2015 | 2015: 12,541 | FOBT: 1 yr; FS: 5 yrs.  Col: 10 yrs | **2008:** 51.6%; **2010:** 58.3%  **2013:** 57.3%; **2015:** 61.3% | **2015:** 50-64: 56.4%  65-75: 71.7% | | **2015:** 60.7% | 62.0% |
| Shapiro (2021)^33^ | NHIS | 2018 | 10595 | FOBT/FIT: 1 yr  Col: 10 yrs; CT col./FS: 5 yrs.; mt-sDNA: 3 yrs | 66.9% (65.8-68.1) | **50-54:** 48.1%; **55-59:** 65.2%  **60-64:** 72.1%; **65-69:** 74.9%; **70-75:** 79.2% | | 67.4% | 66.5% |
|  |  |  |  |  | **2010:** 58.3%; **2013:** 57.3%; **2015:** 61.8% | NR | | NR | |
| Sokale (2022)^42^ | BRFSS**^c^** | 2014-2020 | 779,143 | FOBT/FIT: 1 yr; Col: 10 yrs; FS: 5 yrs+FOBT/FIT-3 yrs. | **2014:** 66.5% (66.1-66.9); **2016:** 67.9% (67.4-68.3); **2018**: 69.6% (69.1-70.1);  **2020:** 72.5% (71.9-73.1) | NR | | NR | |
| Santiago-Rodríguez (2023)^43^ | NHIS | 2010-2018 | 43,624 | FOBT: 1 yr, FS: 5 yrs+ FOBT: 3 yrs, or Col: 10 yrs. | Overall: 62.8% (62.2-63.3)  **US-born:** 64.3 (63.7-64.9); **Foreign-born** (>=15 years): 55.3% (53.8-56.8); **Foreign-born** (<15 years): 35.2% (31.5-39.1) | NR | | NR | |
| He (2023)^35^ | BRFSS**^c^** | 2012-2020 | 2018: 208,540  2020: 175,698 | Col: 10 yrs; FS: 5 yrs+FOBT/FIT:3 yrs.  FOBT/FIT: 1 yr | 2018: 66.7%  2020: 70.4% | NR | | **2018**: 64.7%  **2020:** 69.3% | **2018:** 68.6%  **2020:** 71.4% |
|  | BRFSS**^d^** | 2020 | 175,698 | **Col:** 10 yrs; **FS:** 5-10 yrs; **mt-sDNA:** 3 yrs; **CT col:** 5 yrs; **FIT:** 1 yr | 72.4% | NR | | 71.2% | 73.5% |
| Star (2024)^36^ | NHIS | 2021 | NA | gFOBT/FIT: 1 yr; Col: 10 yrs | 2019: 20.8% (18.9-23.0)  2021: 19.7% 17.8-21.6) | NR | | NR | |

Abbreviations: NHIS, National Health Interview Survey; BRFSS, Behavioral Risk Factor Surveillance System; FOBT, fecal occult blood test; gFOBT, guaiac-based fecal occult blood test; FIT, fecal immunochemical test; mt-sDNA, multi-target stool DNA test; Endo, endoscopy; Col., colonoscopy; FS, flexible sigmoidoscopy; Proct., proctoscopy; NR, not reported; yr(s), year(s).

^a^ Table ordered first according to publication year, then year of data collection. ^b^ Data calculated from the available information in the article. ^c^ Estimate from 2014-2018 was based on the 2008 USPSTF recommendations, which defines up-to-date CRC screening as having had stool blood test within the past year or 3 years, colonoscopy within the past 10 years, sigmoidoscopy within the past 5 years, or blood stool test within the past 3 years. Estimate from 2020 was based on 2018 USPSTF guidelines, and included stool DNA tests in the past 3 years and CT colonography in the past 5 years.^42 d^ Estimate was based on the current 2021 USPSTF recommendations, which defines up-to-date CRC screening as having had (1) a home stool blood test (FOBT or FIT) within 1 year, and/or (2) stool DNA within 3 years, and/or sigmoidoscopy within 5 years with FOBT or FIT within 3 years, and/or (3) colonoscopy within 10 years.^35^

**Table S6.** **Prevalence estimates of recent and lifetime use of fecal test (gFOBT or FIT) in other countries.^a^**

| **First author (Year)** | **Country** | **Data Source** | **Year(s)** | **Sample Size** | **Time frame** | **Overall** | **By Age** | | **Men** | **Women** |
| --- | --- | --- | --- | --- | --- | --- | --- | --- | --- | --- |
| Cardoso (2020)^8^ | Austria | EHIS | 2013-2015 | 125,375 | 2 yrs | 49.0% | NR | | NR | |
|  | Belgium |  |  |  |  | 16.4% |  |  |  |  |
|  | Bulgaria |  |  |  |  | 5.7% |  |  |  |  |
|  | Croatia |  |  |  |  | 22.0% |  |  |  |  |
|  | Cyprus |  |  |  |  | 4.4% |  |  |  |  |
|  | Czechia |  |  |  |  | 42.4% |  |  |  |  |
|  | Denmark |  |  |  |  | 34.8% |  |  |  |  |
|  | Estonia |  |  |  |  | 6.5% |  |  |  |  |
|  | Finland (60-69) |  |  |  |  | 20.1% |  |  |  |  |
|  | France |  |  |  |  | 51.5% |  |  |  |  |
|  | Germany |  |  |  |  | 50.9% |  |  |  |  |
|  | Greece |  |  |  |  | 11.3% |  |  |  |  |
|  | Hungary |  |  |  |  | 13.2% |  |  |  |  |
|  | Iceland |  |  |  |  | 6.0% |  |  |  |  |
|  | Ireland (60-69) |  |  |  |  | 30.6% |  |  |  |  |
|  | Italy (50-69) |  |  |  |  | 31.3% |  |  |  |  |
|  | Latvia |  |  |  |  | 23.1% |  |  |  |  |
|  | Lithuania |  |  |  |  | 22.9% |  |  |  |  |
|  | Luxembourg |  |  |  |  | 24.2% |  |  |  |  |
|  | Malta (55-64) |  |  |  |  | 23.7% |  |  |  |  |
|  | The Netherlands (55-74) |  |  |  |  | 10.1% |  |  |  |  |
|  | Norway |  |  |  |  | 8.2% |  |  |  |  |
|  | Poland |  |  |  |  | 6.6% |  |  |  |  |
|  | Portugal |  |  |  |  | 35.8% |  |  |  |  |
|  | Romania |  |  |  |  | 3.6% |  |  |  |  |
|  | Slovakia |  |  |  |  | 25.9% |  |  |  |  |
|  | Slovenia (50-59) |  |  |  |  | 55.8% |  |  |  |  |
|  | Spain (50-69) |  |  |  |  | 12.4% |  |  |  |  |
|  | Sweden (60-69) |  |  |  |  | 22.5% |  |  |  |  |
|  | UK (60-74) |  |  |  |  | 59.3% |  |  |  |  |
|  | | | | | | | | | | |
| Ola (2024)^10^ | Austria | EHIS | 2018-2020 | 129,750 | 2 yrs | 55.9% | NR | | NR | |
|  | Belgium |  |  |  |  | 36.1% |  |  |  |  |
|  | Bulgaria |  |  |  |  | 3.6% |  |  |  |  |
|  | Croatia |  |  |  |  | 29.2% |  |  |  |  |
|  | Cyprus |  |  |  |  | 3.3% |  |  |  |  |
|  | Czechia |  |  |  |  | 44.7% |  |  |  |  |
|  | Denmark |  |  |  |  | 67.1% |  |  |  |  |
|  | Estonia |  |  |  |  | 21.5% |  |  |  |  |
|  | Finland (60-69) |  |  |  |  | 9.4% |  |  |  |  |
|  | Germany |  |  |  |  | 43.8% |  |  |  |  |
|  | Greece |  |  |  |  | 10.2% |  |  |  |  |
|  | Hungary (50-69) |  |  |  |  | 15.5% |  |  |  |  |
|  | Iceland |  |  |  |  | 6.1% |  |  |  |  |
|  | Ireland (55-74) |  |  |  |  | 33.3% |  |  |  |  |
|  | Italy (50-69) |  |  |  |  | 35.3% |  |  |  |  |
|  | Latvia |  |  |  |  | 25.8% |  |  |  |  |
|  | Lithuania |  |  |  |  | 36.3% |  |  |  |  |
|  | Luxembourg |  |  |  |  | 40.6% |  |  |  |  |
|  | Malta (55-74) |  |  |  |  | 35.7% |  |  |  |  |
|  | The Netherlands (55-74) |  |  |  |  | 64.5% |  |  |  |  |
|  | Norway |  |  |  |  | 8.0% |  |  |  |  |
|  | Poland |  |  |  |  | 7.7% |  |  |  |  |
|  | Portugal |  |  |  |  | 42.2% |  |  |  |  |
|  | Romania |  |  |  |  | 4.2% |  |  |  |  |
|  | Serbia |  |  |  |  | 8.1% |  |  |  |  |
|  | Slovakia |  |  |  |  | 39.3% |  |  |  |  |
|  | Slovenia |  |  |  |  | 54.6% |  |  |  |  |
|  | Spain (50-69) |  |  |  |  | 31.9% |  |  |  |  |
|  | Sweden (60-69) |  |  |  |  | 32.6% |  |  |  |  |
| Major (2015)^56^ | Canada | CCHS | 2008, 2012 | 2012: 9973 | 2 yrs | 2008: 16.9% (16.0-17.8)  2012: 23.0% (22.0-24.0) | **2008:**  50-74: 16.9%  75+: 14.5% | **2012:**  50-74: 23.0%  75+: 18.6% | NR | |
| Singh (2015)^57^ | Canada | CCHS | 2012 | NR. | 2 yrs | 30.1% | **50-54**: 21.2%; **55-59**: 29.4%; **60-64:** 34.7%; **65-69:** 34.6%; **70-74:** 36.4% | | 30.0% | 30.2% |
| Choi (2010)^45^ | Korea | KNCSS | 2005-2008 | 2005: 927  2006: 944  2007: 906  2008: 922 |  | 2005: 7.2%  2006: 13.1%  2007: 17.5%  2008: 21.3% | 2005  50-59: 6.4%  60-79: 7.7%  70-79: 8.4% | 2008  50-59: 19.4%  60-69: 22.5%  70-79: 24.0% | 2005: 8.7%  2008: 21.0% | 2005: 5.9%  2008: 21.6% |
| Park (2012)^46^ | Korea | KNCSS | 2004-2011 | 21865 | 1 yr | **2004:** 3.8%; **2005:** 7.2%  **2006:** 13.6%; **2007:** 20.2%  **2008:** 20.9%; **2009:** 19.0%  **2010:** 25.9%; **2011:** 25.0% | NR | | NR | |
| Suh (2016)^47^ | Korea | KNCSS | 2004-2013 | 30,105 | 1 yr | **2004:** 3.8%; **2005:** 7.2%  **2006:** 13.6%; **2007:** 20.2%  **2008:** 20.9%; **2009:** 19.0%  **2010:** 25.9%; **2011:** 25.0%  **2012:** 29.6%; **2013:** 27.6% | NR | | NR | |
| Bui (2017)^48^ | Korea | KNCSS | 2014 | 2066 | Ever | 52.1% | **50-59:** 55.7%; **60-69**: 32.9%^b^;  **70-74:**11.4%^b^ | | 50.4% | 49.6% |
| Choi (2018)^49^ | Korea | KNCSS | 2013 | 2154 | 1 yr | 29.7% | **50-59:** 60.8%; **60-69**: 27.3%;  **>=70:** 11.9% | | 52.8% | 47.2% |
| Portero de la Cruz (2023)^65^ | Spain | SNHS and EHS | 2017 & 2020 | 2017: 7234  2020: 6929 | 2 yrs | **Overall:** 38.0%  2017: 32.4%  2020: 43.9% | **Overall**  **50-56:** 29.9%; **57-63**: 40.8%  **64-69:** 45.6% | | 37.7% | 38.3% |
| Fedewa (2015)^60^ | Switzerland | SHIS | 2007, 2012 | 2007: 5946  2012: 7224 | 2 yrs | 2007: 13.0%  2012: 9.8% | NR | | NR | |
| Schneider (2022)^61^ | Switzerland | SHIS | 2007-2017 | 2007:5,848  2012:7,335  2017:8,038 | 2 yrs | 2007: 11.1%  2012: 6.7%  2017: 5.3% | 50-59: 4.9%  60-69: 5.7%  70-75: 5.5% | | 5.4% | 5.2% |

### Abbreviations: CCHS, Canadian Community Health Survey; KNCSS, Korean National Cancer Screening Survey; SHIS, Swiss Health Interview Survey; EHIS, European Health Interview Survey**;** SNHS, Spanish National Health Survey; EHS, European Health Survey; gFOBT, guaiac-based fecal occult blood test; FIT, fecal immunochemical test; NR, not reported; yr(s), year(s).

^a^ Table ordered first according to publication year, then the year of data collection.

**Table S7. Prevalence estimates of recent and lifetime use of lower GI endoscopy (Colonoscopy or FS) in other countries.^a^**

| **First author (Yr)** | **Country** | **Data Source** | **Year(s)** | **Sample Size** | **Time frame** | **Overall** | **By Age** | | **Men** | **Women** |
| --- | --- | --- | --- | --- | --- | --- | --- | --- | --- | --- |
| Cardoso (2020)^8^ | Austria | EHIS | 2013-2015 | 125,375 | 10 yrs | 51.6% | NR | | NR | |
|  | Belgium |  |  |  |  | 24.4% |  |  |  |  |
|  | Bulgaria |  |  |  |  | 2.3% |  |  |  |  |
|  | Croatia |  |  |  |  | 14.0% |  |  |  |  |
|  | Cyprus |  |  |  |  | 16.2% |  |  |  |  |
|  | Czechia |  |  |  |  | 24.5% |  |  |  |  |
|  | Denmark |  |  |  |  | 24.9% |  |  |  |  |
|  | Estonia |  |  |  |  | 12.2% |  |  |  |  |
|  | Finland (60-74) |  |  |  |  | 24.2% |  |  |  |  |
|  | France |  |  |  |  | 27.5% |  |  |  |  |
|  | Germany |  |  |  |  | 50.6% |  |  |  |  |
|  | Greece |  |  |  |  | 15.3% |  |  |  |  |
|  | Hungary |  |  |  |  | 15.2% |  |  |  |  |
|  | Iceland |  |  |  |  | 41.1% |  |  |  |  |
|  | Ireland (60-74) |  |  |  |  | 31.9% |  |  |  |  |
|  | Italy |  |  |  |  | 21.7% |  |  |  |  |
|  | Latvia |  |  |  |  | 10.6% |  |  |  |  |
|  | Lithuania |  |  |  |  | 15.7% |  |  |  |  |
|  | Luxembourg |  |  |  |  | 49.0% |  |  |  |  |
|  | Malta (55-69) |  |  |  |  | 15.4% |  |  |  |  |
|  | The Netherlands (55-74) |  |  |  |  | 20.8% |  |  |  |  |
|  | Norway |  |  |  |  | 28.0% |  |  |  |  |
|  | Poland |  |  |  |  | 15.1% |  |  |  |  |
|  | Portugal |  |  |  |  | 35.5% |  |  |  |  |
|  | Romania |  |  |  |  | 4.3% |  |  |  |  |
|  | Slovakia |  |  |  |  | 15.1% |  |  |  |  |
|  | Slovenia |  |  |  |  | 29.0% |  |  |  |  |
|  | Spain |  |  |  |  | 17.6% |  |  |  |  |
|  | Sweden (60-74) |  |  |  |  | 16.7% |  |  |  |  |
|  | UK (60-74) |  |  |  |  | 22.6% |  |  |  |  |
|  | | | | | | | | | | |
| Ola (2024)^10^ | Austria | EHIS | 2018-2020 | 129,750 | 10 yrs | 54.4% | NR | | NR | |
|  | Belgium |  |  |  |  | 27.3% |  |  |  |  |
|  | Bulgaria |  |  |  |  | 4.4% |  |  |  |  |
|  | Croatia |  |  |  |  | 17.9% |  |  |  |  |
|  | Cyprus |  |  |  |  | 18.8% |  |  |  |  |
|  | Czechia |  |  |  |  | 27.1% |  |  |  |  |
|  | Denmark |  |  |  |  | 31.0% |  |  |  |  |
|  | Estonia |  |  |  |  | 19.3% |  |  |  |  |
|  | Finland (60-74) |  |  |  |  | 11.4% |  |  |  |  |
|  | Germany |  |  |  |  | 51.7% |  |  |  |  |
|  | Greece |  |  |  |  | 21.4% |  |  |  |  |
|  | Hungary |  |  |  |  | 17.7% |  |  |  |  |
|  | Iceland |  |  |  |  | 51.5% |  |  |  |  |
|  | Ireland (55-74) |  |  |  |  | 32.8% |  |  |  |  |
|  | Italy |  |  |  |  | 22.2% |  |  |  |  |
|  | Latvia |  |  |  |  | 16.0% |  |  |  |  |
|  | Lithuania |  |  |  |  | 26.5% |  |  |  |  |
|  | Luxembourg (55-74) |  |  |  |  | 60.8% |  |  |  |  |
|  | Malta (55-74) |  |  |  |  | 23.0% |  |  |  |  |
|  | The Netherlands (55-74) |  |  |  |  | 26.9% |  |  |  |  |
|  | Norway |  |  |  |  | 31.4% |  |  |  |  |
|  | Poland |  |  |  |  | 21.9% |  |  |  |  |
|  | Portugal |  |  |  |  | 43.4% |  |  |  |  |
|  | Romania |  |  |  |  | 5.4% |  |  |  |  |
|  | Serbia |  |  |  |  | 9.1% |  |  |  |  |
|  | Slovakia |  |  |  |  | 20.9% |  |  |  |  |
|  | Slovenia |  |  |  |  | 30.1% |  |  |  |  |
|  | Spain |  |  |  |  | 21.5% |  |  |  |  |
|  | Sweden (60-74) |  |  |  |  | 23.9% |  |  |  |  |
| Singh (2015)^57^ | Canada | CCHS | 2012 | NR | FS/Col:10 yrs | 37.2% | **50-54**: 27.0%; **55-59:** 36.4%; **60-64**: 40.7%; **65-69:** 43.5%; **70-74:** 46.5% | | 37.6% | 36.9% |
| Sieverding (2010)^51^ | Germany | HCAP | 2004 | 15,810 | Col: Ever | 35.6% | **50-54**: 25%^b^; **55-59:** 34%^b^  **60-64:** 40%^b^; **65-70:** 43%^b^ | | 33.4%^b^ | 36.8%^b^ |
| Starker (2017)^53^ | Germany | GEDA & EHIS | 2014/2015 | 9,489 | Col: 10 yrs | 58.5% | **Men**  55-59: 48.5%  60-64: 58.5%  65-69: 63.1%  ≥70: 67.5% | **Women**  55-59: 42.8%  60-64: 57.4%  65-69: 64.6%  ≥70: 59.1% | 60.8% | 56.5% |
| Hornschuch (2022)^54^ | Germany | GePaRD | 2008-2017 | 7,475,668 | 10 yrs | 31.0% | **Men**  55-59: 33.2%  60-64: 44.2%  65-69: 49.2%  70-74: 52.6%  75-79: 54.1%  80-84: 51.7% | **Women**  55-59: 35.7%  60-64: 46.5%  65-69: 50.5%  70-74: 52.2%  75-79: 50.9%  80-84: 46.3%. | As reported by age. | |
| Choi (2010)^45^ | Korea | KNCSS | 2005-2008 | 2005: 927; 2006: 944  2007: 906  2008: 922 | Endo: 10 yrs | 2005: 18.0%  2006: 16.5%  2007: 18.0%  2008: 20.5% | **2005: 50-59**: 17.1%; **60-69:** 19.9%  **70-79:** 16.9%; **2006:** NR: **2007:** NR  **2008: 50-59:** 21.3%; **60-69**: 21.5%  **70-79:** 16.6% | | **2005:** 18.2%  **2006:** NR **2007:** NR  **2008:** 19.3% | **2005:** 17.8%  **2006:** NR  **2007:** NR  **2008:** 21.6% |
| Park (2012)^46^ | Korea | KNCSS | 2004-2011 | 2004: 3592 2005: 2028  2006: 2030  2007: 2021  2008: 2038  2009: 2000  2010: 4056  2011: 4100 | Col. 10 yrs | **2004:** 14.4%; **2005:** 12.4%  **2006:** 16.8%; **2007:** 19.5%  **2008:** 19.1%; **2009:** 23.4%  **2010:** 23.3%; **2011**: 23.6% | NR | | NR | |
| Suh (2016)^47^ | Korea | KNCSS | 2004-2013 | 2012: 4,140  2013: 4100 | Col: 10 yrs | 2004-2011: As reported in Park (2012)^46^ | NR | | NR | |
|  |  |  |  |  |  | **2012:** 30.1%; **2013:** 35.2% |  |  |  |  |
| Bui (2017)^48^ | Korea | KNCSS | 2014 | 2066 | Col: Ever | 41.4% | **50-59**: 55%^b^; **60-69**: 35.2%^b^; **70-74**: 9.8%^b^ | | 52.8%^b^ | 47.3%^b^ |
| Fedewa (2015)^60^ | Switzerland | SHIS | 2007, 2012 | 2007: 5946  2012: 7224 | Col: 10 yrs | **2007**: 8.2%  **2012:** 15.0% | NR | | NR | |
| Schneider (2022)^61^ | Switzerland | SHIS | 2007-2017 | 2017: 8,038 | Col: 10 yrs | **2007:** 21.9%; **2012:** 32.5% | NR | | NR | |
|  |  |  |  |  |  | 2017: 42.8% | **50-59:** 35.5%; **60-69:** 47.7%  **70-75:** 52.0% | | 42.8% | 42.7% |

### **Abbreviations:** CCHS, Canadian Community Health Survey; HCAP, Health Care Access Panel; KNCSS, Korean National Cancer Screening Survey; SHIS, Swiss Health Interview Survey; GEDA: Gesundheit in Deutschland aktuell; EHIS, European Health Interview Survey; GePaRD, German Pharmacoepidemiological Research Database; Col., colonoscopy; FS, flexible sigmoidoscopy; NR, not reported; yr(s), year(s).

### ^a^ Table ordered first according to publication year, then the year of data collection.

**Table S8. Prevalence estimates of use of any CRC screening test in other countries.^a^**

| **First author (Yr)** | **Country** | **Data Source** | **Year(s)** | **Sample Size** | **Time frame** | **Overall** | **By Age** | **Men** | **Women** |
| --- | --- | --- | --- | --- | --- | --- | --- | --- | --- |
| Cardoso (2020)^8^ | Austria | EHIS | 2013-2015 | 125,375 | FOBT/FIT: 2 yrs  Col. 10 yrs. | 68.8% | NR | NR | |
|  | Belgium |  |  |  |  | 32.7% |  |  |  |
|  | Bulgaria |  |  |  |  | 6.9% |  |  |  |
|  | Croatia |  |  |  |  | 29.7% |  |  |  |
|  | Cyprus |  |  |  |  | 17.9% |  |  |  |
|  | Czechia |  |  |  |  | 53.5% |  |  |  |
|  | Denmark |  |  |  |  | 47.0% |  |  |  |
|  | Estonia |  |  |  |  | 16.1% |  |  |  |
|  | Finland (60-74) |  |  |  |  | 34.8% |  |  |  |
|  | France |  |  |  |  | 60.1% |  |  |  |
|  | Germany |  |  |  |  | 70.9% |  |  |  |
|  | Greece |  |  |  |  | 22.7% |  |  |  |
|  | Hungary |  |  |  |  | 23.0% |  |  |  |
|  | Iceland |  |  |  |  | 42.2% |  |  |  |
|  | Ireland (60-74) |  |  |  |  | 42.7% |  |  |  |
|  | Italy |  |  |  |  | 41.4% |  |  |  |
|  | Latvia |  |  |  |  | 28.4% |  |  |  |
|  | Lithuania |  |  |  |  | 27.7% |  |  |  |
|  | Luxembourg |  |  |  |  | 55.6% |  |  |  |
|  | Malta (55-69) |  |  |  |  | 30.6% |  |  |  |
|  | The Netherlands (55-74) |  |  |  |  | 24.9% |  |  |  |
|  | Norway |  |  |  |  | 30.5% |  |  |  |
|  | Poland |  |  |  |  | 19.1% |  |  |  |
|  | Portugal |  |  |  |  | 55.4% |  |  |  |
|  | Romania |  |  |  |  | 6.3% |  |  |  |
|  | Slovakia |  |  |  |  | 33.4% |  |  |  |
|  | Slovenia |  |  |  |  | 65.0% |  |  |  |
|  | Spain |  |  |  |  | 25.4% |  |  |  |
|  | Sweden (60-74) |  |  |  |  | 28.0% |  |  |  |
|  | UK (60-74) |  |  |  |  | 66.7% |  |  |  |
|  | | | | | | | | | |
| Ola (2024)^10^ | Austria | EHIS | 2018-2020 | 129,750 | FOBT/FIT: 2 yrs  Col. 10 yrs. | 72.8% | NR | NR | |
|  | Belgium |  |  |  |  | 51.7% |  |  |  |
|  | Bulgaria |  |  |  |  | 6.3% |  |  |  |
|  | Croatia |  |  |  |  | 37.7% |  |  |  |
|  | Cyprus |  |  |  |  | 20.9% |  |  |  |
|  | Czechia |  |  |  |  | 55.9% |  |  |  |
|  | Denmark |  |  |  |  | 74.9% |  |  |  |
|  | Estonia |  |  |  |  | 31.9% |  |  |  |
|  | Finland (60-74) |  |  |  |  | 16.7% |  |  |  |
|  | Germany |  |  |  |  | 68.8% |  |  |  |
|  | Greece |  |  |  |  | 26.2% |  |  |  |
|  | Hungary |  |  |  |  | 26.8% |  |  |  |
|  | Iceland |  |  |  |  | 52.6% |  |  |  |
|  | Ireland (55-74) |  |  |  |  | 49.7% |  |  |  |
|  | Italy (50-74) |  |  |  |  | 45.3% |  |  |  |
|  | Latvia |  |  |  |  | 34.2% |  |  |  |
|  | Lithuania |  |  |  |  | 48.0% |  |  |  |
|  | Luxembourg (55-74) |  |  |  |  | 75.4% |  |  |  |
|  | Malta (55-74) |  |  |  |  | 48.4% |  |  |  |
|  | The Netherlands (55-74) |  |  |  |  | 73.2% |  |  |  |
|  | Norway |  |  |  |  | 33.6% |  |  |  |
|  | Poland |  |  |  |  | 24.9% |  |  |  |
|  | Portugal |  |  |  |  | 65.9% |  |  |  |
|  | Romania |  |  |  |  | 8.1% |  |  |  |
|  | Slovakia |  |  |  |  | 45.9% |  |  |  |
|  | Serbia |  |  |  |  | 13.6% |  |  |  |
|  | Slovenia |  |  |  |  | 68.2% |  |  |  |
|  | Spain |  |  |  |  | 42.8% |  |  |  |
|  | Sweden (60-74) |  |  |  |  | 38.1% |  |  |  |
| Singh (2015)^57^ | Canada | CCHS | 2012 | NR | Either or both FOBT (1 yr) and FS/Col. | 55.2% | **50-54:** 41.8%; **55-59**: 54.7%; **60-64**: 61.0%; **65-69:** 62.5%; **70-74**: 65.3% | 54.9% | 55.5% |
| Choi (2010)^45^ | Korea | KNCSS | 2005-2008 | 2005: 927  2006: 944  2007: 906  2008: 922 | Col: 10 yrs, DCBE: 5 yrs; FOBT: 1 yr | 2005: 22.9%  2006: 26.8%  2007: 29.0%  2008: 36.6% | NR | NR | |
| Park (2012)^46^ | Korea | KNCSS | 2004-2011 | 21,865 | Col: 10 yrs, FOBT: 1 yr; DCBE: 5 yrs | As reported in Suh (2016)^47^ | As reported in Suh (2016)^53^ | As reported in Suh (2016)^53^ | |
| Suh (2016)^47^ | Korea | KNCSS | 2004-2013 | 30,105 | Col: 10 yrs, FOBT: 1 yr; DCBE: 5 yrs | 2004: 19.9% | **50-59:** 19.4%; **60-69:** 21.9%; **≥ 70:** 17.6% | 21.5% | 18.5% |
|  |  |  |  |  |  | 2005: 25.4% | **50-59**: 23.5%; **60-69:** 28.1%; **≥ 70:** 29.7% | 28.0% | 23.0% |
|  |  |  |  |  |  | 2006: 29.4% | **50-59:** 26.0%; **60-69:** 37.1%; **≥ 70:** 18.6% | 31.2% | 27.8% |
|  |  |  |  |  |  | 2007: 34.1% | **50-59:** 30.7%; **60-69**: 38.5%; **≥ 70:** 29.1% | 36.9% | 31.8% |
|  |  |  |  |  |  | 2008: 37.9% | **50-59:** 40.0%; **60-69:** 40.7%; **≥ 70:** 35.6% | 39.3% | 40.1% |
|  |  |  |  |  |  | 2009: 36.7% | **50-59:** 35.5%; **60-69:** 38.7%; **≥ 70:** 35.9% | 36.5% | 36.8% |
|  |  |  |  |  |  | 2010: 35.5% | **50-59:** 33.3%; **60-69:** 38.4%; **≥ 70:** 37.3% | 38.5% | 32.7% |
|  |  |  |  |  |  | 2011: 35.3% | **50-59**: 32.7%; **60-69:** 38.3%; **≥ 70:** 39.6% | 37.4% | 33.3% |
|  |  |  |  |  |  | 2012: 44.7% | **50-59:** 42.9%; **60-69:** 48.0%; **≥ 70:** 44.4% | 46.7% | 42.8% |
|  |  |  |  |  |  | 2013: 55.6% | **50-59:** 54.7%; **60-69:** 57.4%; **≥ 70:** 55.0% | 56.3% | 54.9% |
| Choi (2018)^49^ | Korea | KNCSS | 2013 | 2154 | FOBT: 1 yr, Col: 10 yrs. | 67.0% | **50-59:** 56.5%; **60-69**: 32.1%; **>=70**: 11.4% | 49.6% | 50.4% |
| Park (2022)^50^ | Korea | KNCSS**^b^** | 2005-2020 | 29,040 | FIT: 1 yr, DCBE: 5 yrs, or col.: 10 yrs (2005–2018) &  FIT- 1 yr or col.: 10 yrs (2019–2020) | 2005: 25.0% | **50-59:** 22.9%; **60-69**: 28.7%; **70-74**: 25.9% | 27.2% | 23.1% |
|  |  |  |  |  |  | 2006: 29.4% | **50-59:** 26.2%; **60-69:** 37.1%; **70-74:** 18.6% | 31.2% | 27.9% |
|  |  |  |  |  |  | 2007: 34.1% | **50-59:** 30.7%; **60-69:** 38.3%; **70-74:** 29.0% | 36.7% | 31.9% |
|  |  |  |  |  |  | 2008: 39.8% | **50-59:** 40.2%; **60-69:** 40.7%; **70-74:** 35.3% | 39.4% | 40.1% |
|  |  |  |  |  |  | 2009: 36.7% | **50-59:** 35.5%; **60-69:** 38.8%; **70-74:** 35.6% | 36.5% | 36.8% |
|  |  |  |  |  |  | 2010: 35.5% | **50-59:** 33.3%; **60-69:** 38.4%; **70-74:** 36.9% | 38.6% | 32.5% |
|  |  |  |  |  |  | 2011: 35.3% | **50-59:** 32.7%; **60-69:** 38.3%; **70-74:** 39.6% | 37.4% | 33.3% |
|  |  |  |  |  |  | 2012: 44.7% | **50-59**: 42.9%; **60-69:** 48.0%; **70-74**: 44.4% | 46.7% | 42.8% |
|  |  |  |  |  |  | 2013: 55.6% | **50-59:** 54.7%; **60-69**: 57.4%; **70-74:** 55.0% | 56.3% | 54.9% |
|  |  |  |  |  |  | 2014: 60.1% | **50-59:** 59.0%; **60-69:** 63.3%; **70-74**: 56.4% | 64.1% | 56.2% |
|  |  |  |  |  |  | 2015: 59.5% | **50-59:** 59.0%; **60-69:** 62.9%; **70-74:** 52.6% | 64.2% | 55.0% |
|  |  |  |  |  |  | 2016: 54.6% | **50-59:** 53.8%; **60-69:** 55.7%; **70-74**: 55.0% | 55.3% | 53.9% |
|  |  |  |  |  |  | 2017: 56.8% | **50-59:** 56.9%; **60-69:** 57.2%; **70-74:** 54.6% | 60.9% | 52.7% |
|  |  |  |  |  |  | 2018: 58.4% | **50-59:** 58.6%; **60-69:** 59.6%; **70-74:** 53.8% | 60.8% | 56.1% |
|  |  |  |  |  |  | 2019: 62.6% | **50-59**: 61.2%; **60-69:** 66.2%; **70-74:** 59.8% | 63.8% | 61.5% |
|  |  |  |  |  |  | 2020: 64.4% | **50-59:** 63.4%; **60-69**: 68.0%; **70-74:** 60.3% | 67.3% | 61.6% |
| Khoja (2018)^63^ | Saudi Arabia | SNSEH | 2006-2007 | 2,946 | FOBT: 1 yr  Col: 5 yrs. | 5.6% | **60-65**: 5.9%; **66-70**: 6%; **71-75**: 6.6%; **76-80**: 3.3% **81-85**: 6.2%; **86-90**: 2.6%; **>90**: 5.8% | 5.9%^b^ | 5.4%^b^ |
| Schneider (2022)^61^ | Switzerland | SHIS | 2007-2017 | **2007**: 5,848  **2012:** 7,335  **2017**: 8,038 | Any test | 2007: 33.0%  2012: 39.2%  2017: 48.1% | 2017: **50-59:** 40.4%; **60-69:** 53.4%;  **70-75:** 57.6% | 2017: 48.2% | 47.9% |

Abbreviations: CCHS, Canadian Community Health Survey; KNCSS, Korean National Cancer Screening Survey; SHIS, Swiss Health Interview Survey; SNSEH, Saudi National Survey for Elderly Health; EHIS, European Health Interview Survey; FOBT, fecal occult blood test; FIT, fecal immunochemical test; Col., colonoscopy; FS, flexible sigmoidoscopy; DCBE, double-contrast barium enema; NR, not reported; yr(s), year(s).

^a^ Table ordered first according to publication year, then the year of data collection. ^b^ Up-to-date with CRC screening was defined as FIT within 1 year, or DCBE within 5 years, or colonoscopy within 10 years in 2005–2018. This was changed to FIT within 1 year or colonoscopy within 10 years in 2019–2020.^50^
