# Supplementary material for: Utilization of colorectal cancer screening tests: a systematic review and time trend analysis of nationally representative data
Source: eClinicalMedicine. 2024 Aug 21;75:102783. doi: 10.1016/j.eclinm.2024.102783 (PMC11388351; doi:10.1016/j.eclinm.2024.102783)
Supplement: Supplementary Appendix 2 [file mmc2.docx]

**Utilization of Colorectal Cancer Screening Tests:**

**A Systematic Review and Time Trend Analysis of Nationally Representative Data**

**Idris Ola ^1, 2^ Rafael Cardoso^1^ Michael Hoffmeister ^1^ Hermann Brenner ^1, 3, 4^**

## **SUPPLEMENTARY APPENDIX 2**

**Table of contents**

Page no.

1. Title and table of contents 1
2. Table E1. Articles excluded with reasons after full text review. 2-4
3. Item E2. Reference list of excluded articles. 5-16

| **Table E1. Articles excluded with reasons after full text review**. | | | | |
| --- | --- | --- | --- | --- |
|  | | | | |
| **Category of reasons for exclusion** | **Articles excluded (First author, year)^a^** | **Country** | **Additional Notes** | **Reference** |
| **No relevant outcome** | Winawer (1982) | US | NA | ^1^ |
|  | Schroy (1999) |  | No national representativeness, no relevant study population. | ^2^ |
|  | Lieberman (2000) |  | NA | ^3^ |
|  | Weissfeld (2002) |  |  | ^4^ |
|  | Crosswell (2009) |  |  | ^5^ |
|  | Sequist (2009) |  | No national representativeness, no relevant study population. | ^6^ |
|  | Walsh (2010) |  | No national representativeness | ^7^ |
|  | Schoen (2010) |  | No national representativeness and program evaluation metrics. | ^8^ |
|  | Goodwin (2011) |  | NA | ^9^ |
|  | Curry (2011) |  | No national representativeness | ^10^ |
|  | Redwood (2012) |  | No national representativeness | ^11^ |
|  | Rogal (2012) |  | NA | ^12^ |
|  | Dorn (2012) |  | No national representativeness | ^13^ |
|  | Hawley (2012) |  |  | ^14^ |
|  | Inadomi (2012) |  |  | ^15^ |
|  | Arnold (2012) |  |  | ^16^ |
|  | Laiyemo (2012) |  | NA | ^17^ |
|  | Weiss (2013) |  | No national representativeness, no relevant study population. | ^18^ |
|  | Cooper (2013) |  | No national representativeness | ^19^ |
|  | Saini (2014) |  |  | ^20^ |
|  | Charlton (2014) |  |  | ^21^ |
|  | Gupta (2014) |  | No national representativeness, no relevant study population. | ^22^ |
|  | Davis (2014) |  | No national representativeness | ^23^ |
|  | Son (2014) |  |  | ^24^ |
|  | Wernli (2014) |  |  | ^25^ |
|  | Collazo (2015) |  |  | ^26^ |
|  | Saini (2016) |  |  | ^27^ |
|  | Gupta (2016) |  |  | ^28^ |
|  | Singal (2016) |  |  | ^29^ |
|  | Halm (2016) |  | NA | ^30^ |
|  | Green (2016) |  |  | ^31^ |
|  | Arnold (2016) |  | No national representativeness | ^32^ |
|  | Doroudi (2017) |  | NA | ^33^ |
|  | Davis (2017) |  | No national representativeness and program evaluation metrics. | ^34^ |
|  | Singal (2017) |  | No national representativeness | ^35^ |
|  | Preston (2018) |  | No national representativeness | ^36^ |
|  | Huei-Yu Wang (2018) |  | No national representativeness, no relevant study population. | ^37^ |
|  | Hughes (2018) |  | No national representativeness | ^38^ |
|  | Alsayid (2018) |  | NA | ^39^ |
|  | Green (2019) |  |  | ^40^ |
|  | Pierre-Victor (2019) |  |  | ^41^ |
|  | Nielson (2019) |  |  | ^42^ |
|  | Champion (2020) |  | No national representativeness | ^43^ |
|  | Somsouk (2020) |  | NA | ^44^ |
|  | Haverkamp (2020) |  | No national representativeness | ^45^ |
|  | O'Connor (2020) |  | NA | ^46^ |
|  | Lucas (2021) |  | No national representativeness | ^47^ |
|  | Huf (2021) |  |  | ^48^ |
|  | Coronado (2021) |  |  | ^49^ |
|  | Shepherd (2021) |  |  | ^50^ |
|  | Green (2022) |  |  | ^51^ |
|  | Oyalowo (2022) |  |  | ^52^ |
|  | Goshgarian (2022) |  |  | ^53^ |
|  | Liang (2022) |  | NA | ^54^ |
|  | O’Leary (2023) |  | No national representativeness | ^55^ |
|  | Miller (2024) |  | Full text not accessible | ^56^ |
|  | **Other countries** | | | |
|  | Dodd (2019) | Australia | No national representativeness | ^57^ |
|  | Hoeck (2019) | Belgium |  | ^58^ |
|  | Tinmouth (2014) | Canada |  | ^59^ |
|  | López-Kostner (2018) | Chile |  | ^60^ |
|  | Zhan (2019) | China | No national representativeness and program evaluation metrics. | ^61^ |
|  | Jørgensen (2002) | Denmark | No national representativeness and program evaluation metrics. | ^62^ |
|  | Allam (2024) | Egypt | Study conducted among potentially high-risk participants | ^63^ |
|  | Bretthauer (2016) | Europe | Program evaluation metrics. | ^64^ |
|  | Murphy (2019) | Europe | NA | ^65^ |
|  | Rat (2017) | France | No national representativeness, no relevant study population. | ^66^ |
|  | Denis (2007) | France | No national representativeness and program evaluation metrics. | ^67^ |
|  | Mansmann (2008) | Germany | No national representativeness | ^68^ |
|  | Brenner (2017) |  |  | ^69^ |
|  | Hoffmeister (2019) |  |  | ^70^ |
|  | Carr (2020) |  | NA | ^71^ |
|  | Gruner (2020) |  | No national representativeness | ^72^ |
|  | Heisser (2020) |  | NA | ^73^ |
|  | Schwarz (2020) |  |  | ^74^ |
|  | Wong (2014) | Hong Kong | Program evaluation metrics. | ^75^ |
|  | Wong (2018) |  | NA | ^76^ |
|  | Purnomo (2023) | Indonesia | No national representativeness | ^77^ |
|  | Segnan (2011) | Italy | NA | ^78^ |
|  | Yamaji (2004) | Japan | No national representativeness and program evaluation metrics. | ^79^ |
|  | Hoff (2008) | Norway | No national representativeness | ^80^ |
|  | Hoff (2009) |  |  | ^81^ |
|  | García-Albéniz (2017) |  | NA | ^82^ |
|  | Pisera (2019) | Poland |  | ^83^ |
|  | Guiriguet (2016) | Spain | No national representativeness | ^84^ |
|  | Valiente González (2021) |  | No national representativeness | ^85^ |
|  | Chua (2014) | Singapore | NA | ^86^ |
|  | Chiu (2015) | Taiwan |  | ^87^ |
|  | Verne (1998) | UK | No national representativeness and program evaluation metrics. | ^88^ |
|  | Atkin (1998) |  | No national representativeness and program evaluation metrics. | ^89^ |
|  | Scholefield (2002) |  | No national representativeness | ^90^ |
|  | Atkin (2010) |  | NA | ^91^ |
|  | Morris (2015) |  |  | ^92^ |
|  | Raine (2016) |  |  | ^93^ |
|  | Raine (2016) |  |  | ^94^ |
|  | Atkin (2017) |  |  | ^95^ |
|  | Smith (2017) |  |  | ^96^ |
|  | | | | |
| **Participation/attendance rates and other screening outcome metrics** | Costas-Muñiz (2016) | US | No national representativeness | ^97^ |
|  | Murphy (2019) |  |  | ^98^ |
|  | Miller-Wilson (2021) |  |  | ^99^ |
|  | **Other countries** | | | |
|  | Liu (2024) | China | No national representativeness | ^100^ |
|  | Yu (2024) |  | No relevant outcome | ^101^ |
|  | Dong (2024) |  |  | ^102^ |
|  | Gram (2021) | Denmark | NA | ^103^ |
|  | Pellat (2018) | France |  | ^104^ |
|  | Hoffmeister (2017) | Germany | No national representativeness | ^105^ |
|  | Zorzi (2006) | Italy | NA | ^106^ |
|  | Zorzi (2011) |  |  | ^107^ |
|  | Zorzi (2012) |  |  | ^108^ |
|  | Zorzi (2015) |  |  | ^109^ |
|  | Saito (2021) | Japan | No national representativeness | ^110^ |
|  | Rim (2017) | Korea | NA | ^111^ |
|  | Santare (2015) | Latvia |  | ^112^ |
|  | Dulskas (2021) | Lithuania |  | ^113^ |
|  | Tamin (2020) | Malaysia |  | ^114^ |
|  | Quyn (2018) | Scotland |  | ^115^ |
|  | Cheng (2021) | Taiwan | No national representativeness | ^116^ |
|  | UK Colorectal Cancer Screening Pilot Group (2004) | UK | No national representativeness | ^117^ |
|  | Steele (2010) |  | NA | ^118^ |
|  | Lo (2012) |  | No national representativeness | ^119^ |
|  | Logan (2012) |  | NA | ^120^ |
|  | Lo (2015) |  |  | ^121^ |
|  | | | | |
| **Background article** | Kaminski (2012) | Poland | NA | ^122^ |
|  | Kaminski (2015) | Poland |  | ^123^ |
|  | Hirst (2016) | UK |  | ^124^ |
|  | | | | |
| **Study conducted among high-risk group** | Cooper (2015) | US | NA | ^125^ |
|  | | | | |
| **Data already provided by other studies** | Doubeni (2011) | US | NA | ^126^ |
|  | Tak (2022) |  |  | ^127^ |
|  | Liu (2023) |  |  | ^128^ |
|  | **Other countries** | | | |
|  | Shim (2010) | Korea | NA | ^129^ |
|  | | | | |
| **Study population not clearly defined (e.g age group <50 years included in analysis)** | Koo (2012) | Asia | No national representativeness | ^130^ |
|  | Ansa (2024) | US | NA | ^131^ |
|  | | | | |
| **No national representativeness** | Walsh (2004) | US | NA | ^132^ |
|  | Ko (2005) |  |  | ^133^ |
|  | Thompson (2005) |  |  | ^134^ |
|  | Farmer (2008) |  |  | ^135^ |
|  | McNeill (2009) |  |  | ^136^ |
|  | Walter (2009) |  |  | ^137^ |
|  | Maxwell (2009) |  |  | ^138^ |
|  | Adams-Campbell (2010) |  |  | ^139^ |
|  | Cokkinides (2011) |  |  | ^140^ |
|  | Day (2011) |  |  | ^141^ |
|  | Khatami (2012) |  |  | ^142^ |
|  | Sinicrope (2012) |  |  | ^143^ |
|  | Doubeni (2012) |  |  | ^144^ |
|  | Cohen (2012) |  |  | ^145^ |
|  | May (2014) |  |  | ^146^ |
|  | Halpern (2014) |  |  | ^147^ |
|  | Wilcox (2015) |  |  | ^148^ |
|  | Kotwal (2016) |  |  | ^149^ |
|  | Richman (2016) |  |  | ^150^ |
|  | Waghray (2016) |  |  | ^151^ |
|  | Siantz (2017) |  |  | ^152^ |
|  | Ghai (2018) |  |  | ^153^ |
|  | Buehler (2019) |  |  | ^154^ |
|  | Bhandari (2019) |  |  | ^155^ |
|  | Rastogi (2019) |  |  | ^156^ |
|  | Viramontes (2020) |  |  | ^157^ |
|  | Alyabsi (2020) |  |  | ^158^ |
|  | Ng (2021) |  |  | ^159^ |
|  | McEvoy (2021) |  |  | ^160^ |
|  | Ansa (2021) |  |  | ^161^ |
|  | Fisher (2021) |  |  | ^162^ |
|  | Fisher (2022) |  |  | ^163^ |
|  | Kowalkowski (2023) |  |  | ^164^ |
|  | **Other countries** | | | |
|  | Hermann (2015) | Germany | NA | ^165^ |
|  | Mohammad (2024) | Iraq |  | ^166^ |
|  | Sharma (2021) | Nigeria |  | ^167^ |
|  | Thulin (2021) | Sweden |  | ^168^ |

## ^a^ Table ordered first according to country, then publication year. The US (United States) was placed as the first country in every category due to the large number of articles from the country. NA, not applicable.

## **Item E2. Reference list of excluded articles**

1. Winawer SJ, Fleisher M, Baldwin M, Sherlock P. Current status of fecal occult blood testing in screening for colorectal cancer. *CA Cancer J Clin*. 1982;**32**:100-12. doi:10.3322/canjclin.32.2.100
2. Schroy PC, Heeren T, Bliss CM, *et al*. Implementation of on-site screening sigmoidoscopy positively influences utilization by primary care providers. *Gastroenterol*. 1999;**117**:304-11. doi:10.1053/gast.1999.0029900304.
3. Lieberman DA, Weiss DG, Bond JH, et al. Use of colonoscopy to screen asymptomatic adults for colorectal cancer. Veterans Affairs Cooperative Study Group 380 [published correction appears in N Engl J Med 2000 Oct 19;343(16):1204]. *N Engl J Med*. 2000;**343**:162-8. doi:10.1056/NEJM200007203430301
4. Weissfeld JL, Ling BS, Schoen RE, et al. Adherence to repeat screening flexible sigmoidoscopy in the Prostate, Lung, Colorectal, and Ovarian (PLCO) Cancer Screening Trial. *Cancer*. 2002;**94**:2569-2576. doi:10.1002/cncr.10538
5. Croswell JM, Kramer BS, Kreimer AR, et al. Cumulative incidence of false-positive results in repeated, multimodal cancer screening. *Ann Fam Med*. 2009;**7**:212-222. doi:10.1370/afm.942
6. Sequist TD, Zaslavsky AM, Marshall R, et al. Patient and physician reminders to promote colorectal cancer screening: a randomized controlled trial. *Arch Intern Med*. 2009;**169**:364-71. doi:10.1001/archinternmed.2008.564
7. Walsh JM, Salazar R, Nguyen TT, et al. Healthy colon, healthy life: a novel colorectal cancer screening intervention. *Am J Prev Med*. 2010;**39**:1-14. doi:10.1016/j.amepre.2010.02.020
8. Schoen RE, Pinsky PF, Weissfeld JL, et al. Utilization of surveillance colonoscopy in community practice. *Gastroenterol*. 2010;**138**:73-81. doi:10.1053/j.gastro.2009.09.062.
9. Goodwin JS, Singh A, Reddy N, et al. Overuse of screening colonoscopy in the medicare population. *Arch Intern Med.* 2011;**171**:1335–43. doi:10.1001/archinternmed.2011.212
10. Curry WJ, Lengerich EJ, Kluhsman BC, et al. Academic detailing to increase colorectal cancer screening by primary care practices in Appalachian Pennsylvania. *BMC Health Serv Res.* 2011;**23**:112. doi:10.1186/1472-6963-11-112.
11. Redwood D, Provost E, Perdue D, et al. The last frontier: innovative efforts to reduce colorectal cancer disparities among the remote Alaska Native population. *Gastrointest Endosc*. 2012;**75**:474-80. doi:10.1016/j.gie.2011.12.031
12. Rogal SS, Pinsky PF, Schoen RE. Relationship between detection of adenomas by flexible sigmoidoscopy and interval distal colorectal cancer. *Clin Gastroenterol Hepatol.* 2013;**11**:73-8. doi: 10.1016/j.cgh.2012.08.002.
13. Dorn SD, Wei D, Farley JF, et al. Impact of the 2008-2009 economic recession on screening colonoscopy utilization among the insured. *Clin Gastroenterol Hepatol.* 2012;**10**:278-84. doi: 10.1016/j.cgh.2011.11.020.
14. Hawley ST, McQueen A, Bartholomew LK, et al. Preferences for colorectal cancer screening tests and screening test use in a large multispecialty primary care practice. *Cancer*. 2012;**118**:2726-34. doi: 10.1002/cncr.26551.
15. Inadomi JM, Vijan S, Janz NK, et al. Adherence to colorectal cancer screening: a randomized clinical trial of competing strategies. *Arch Intern Med*. 2012;**172**:575-582. doi:10.1001/archinternmed.2012.332
16. Arnold CL, Rademaker A, Bailey SC, et al. Literacy barriers to colorectal cancer screening in community clinics. *J Health Commun*. 2012;**17** Suppl 3(0 3):252-64. doi:10.1080/10810730.2012.713441
17. Laiyemo AO, Doubeni C, Pinsky PF, et al. Factors associated with inadequate colorectal cancer screening with flexible sigmoidoscopy. *Cancer Epidemiol*. 2012;**36**:395-9. doi:10.1016/j.canep.2011.10.013.
18. Weiss JM, Smith MA, Pickhardt PJ, et al. Predictors of colorectal cancer screening variation among primary-care providers and clinics. *Am J Gastroenterol*. 2013;**108**:1159-67. doi:10.1038/ajg.2013.127
19. Cooper GS, Kou TD, Barnholtz Sloan JS, et al. Use of colonoscopy for polyp surveillance in Medicare beneficiaries. *Cancer*. 2013;**119**:1800-07. doi:10.1002/cncr.27990
20. Saini SD, Vijan S, Schoenfeld P, et al. Role of quality measurement in inappropriate use of screening for colorectal cancer: retrospective cohort study. *BMJ*. 2014;**348**:g1247. doi:10.1136/bmj.g1247.
21. Charlton ME, Mengeling MA, Halfdanarson TR, et al. Evaluation of a home-based colorectal cancer screening intervention in a rural state. *J Rural Health*. 2014;**30**:322-32. doi:10.1111/jrh.12052
22. Gupta S, Brenner AT, Ratanawongsa N, Inadomi JM. Patient trust in physician influences colorectal cancer screening in low-income patients. *Am J Prev Med*. 2014;**47**:417-23. doi:10.1016/j.amepre.2014.04.020
23. Davis TC, Arnold CL, Bennett CL, et al. Strategies to improve repeat fecal occult blood testing cancer screening. *Cancer Epidemiol Biomarkers Prev*. 2014;**23**:134-43. doi:10.1158/1055-9965.EPI-13-0795
24. Son P, Lane DS, Messina CR, *et al.* Impact of project SCOPE on racial/ethnic disparities in screening colonoscopies. *J. Racial and Ethnic Health Disparities* 2014;**1**:110–19. <https://doi.org/10.1007/s40615-014-0016-4>
25. Wernli KJ, Hubbard RA, Johnson E, et al. Patterns of colorectal cancer screening uptake in newly eligible men and women. *Cancer Epidemiol Biomarkers Prev*. 2014;**23**:1230-7. doi:10.1158/1055-9965.EPI-13-1360.
26. Collazo TH, Jandorf L, Thelemaque L, et al. Screening colonoscopy among uninsured and underinsured urban minorities. *Gut Liver*. 2015;**9**:502-8. doi:10.5009/gnl14039.
27. Saini SD, Powell AA, Dominitz JA, et al. Developing and testing an electronic measure of screening colonoscopy overuse in a large integrated healthcare system. *J Gen Intern Med*. 2016;**31** Suppl 1(Suppl 1):53-60. doi:10.1007/s11606-015-3569-y
28. Gupta S, Miller S, Koch M, et al. Financial Incentives for Promoting Colorectal Cancer Screening: A Randomized, Comparative Effectiveness Trial. *Am J Gastroenterol*. 2016;**111**:1630-6. doi:10.1038/ajg.2016.286
29. Singal AG, Gupta S, Tiro JA, et al. Outreach invitations for FIT and colonoscopy improve colorectal cancer screening rates: A randomized controlled trial in a safety-net health system. *Cancer*. 2016;**122**:456-63. doi:10.1002/cncr.29770
30. Halm EA, Beaber EF, McLerran D, et al. Association between primary care visits and colorectal cancer screening outcomes in the era of population health outreach. *J Gen Intern Med.* 2016;**31**:1190-7. doi: 10.1007/s11606-016-3760-9.
31. Green BB, Anderson ML, Chubak J, et al. Impact of continued mailed fecal tests in the patient-centered medical home: Year 3 of the Systems of Support to Increase Colon Cancer Screening and Follow-Up randomized trial. *Cancer*. 2016;**122**:312-21. doi: 10.1002/cncr.29734.
32. Arnold CL, Rademaker A, Wolf MS, et al. Third annual fecal occult blood testing in community health clinics. *Am J Health Behav*. 2016;**40**:302-9. doi:10.5993/AJHB.40.3.2
33. Doroudi M, Schoen RE, Pinsky PF. Early detection versus primary prevention in the PLCO flexible sigmoidoscopy screening trial: Which has the greatest impact on mortality?. *Cancer*. 2017;**123**:4815-22. doi:10.1002/cncr.31034
34. Davis SN, Christy SM, Chavarria EA, et al. A randomized controlled trial of a multicomponent, targeted, low-literacy educational intervention compared with a nontargeted intervention to boost colorectal cancer screening with fecal immunochemical testing in community clinics. *Cancer*. 2017;**123**:1390-1400. doi:10.1002/cncr.30481
35. Singal AG, Gupta S, Skinner CS, et al. Effect of Colonoscopy Outreach vs Fecal Immunochemical Test Outreach on Colorectal Cancer Screening Completion: A Randomized Clinical Trial. *JAMA*. 2017;**318**:806-15. doi:10.1001/jama.2017.11389
36. Preston MA, Glover-Collins K, Ross L, et al. Colorectal cancer screening in rural and poor-resourced communities. *Am J Surg*. 2018;**216**:245-50. doi:10.1016/j.amjsurg.2017.08.004
37. Huei-Yu Wang J, Ma GX, Liang W, et al. Physician Intervention and Chinese Americans' Colorectal Cancer Screening. *Am J Health Behav*. 2018;**42**:13-26. doi:10.5993/AJHB.42.1.2
38. Hughes AE, Tiro JA, Balasubramanian BA, et al. Social disadvantage, healthcare utilization, and colorectal cancer screening: Leveraging longitudinal patient address and health records data. *Cancer Epidemiol Biomarkers Prev*. 2018;**27**:1424-32. doi:10.1158/1055-9965.EPI-18-0446
39. Alsayid M, Singh MH, Issaka R, et al. Yield of colonoscopy after a positive result from a fecal immunochemical test OC-light. *Clin Gastroenterol Hepatol*. 2018;**16**:1593-97.e1. doi:10.1016/j.cgh.2018.04.014.
40. Green BB, Anderson ML, Cook AJ, et al. Financial incentives to increase colorectal cancer screening uptake and decrease disparities: A randomized clinical trial. *JAMA Netw Open*. 2019;**2**:e196570. doi:10.1001/jamanetworkopen.2019.6570
41. Pierre-Victor D, Pinsky PF. Association of nonadherence to cancer screening examinations with mortality from unrelated causes: A secondary analysis of the PLCO cancer screening trial. *JAMA Intern Med*. 2019;**179**:196-203. doi:10.1001/jamainternmed.2018.5982
42. Nielson CM, Vollmer WM, Petrik AF, et al. Factors affecting adherence in a pragmatic trial of annual fecal immunochemical testing for colorectal cancer. *J Gen Intern Med*. 2019;**34**:978-85. doi:10.1007/s11606-018-4820-0
43. Champion VL, Christy SM, Rakowski W, et al. An RCT to increase breast and colorectal cancer screening. *Am J Prev Med*. 2020;**59**:e69-e78. doi:10.1016/j.amepre.2020.03.008
44. Somsouk M, Rachocki C, Mannalithara A, et al. Effectiveness and cost of organized outreach for colorectal cancer screening: a randomized, controlled trial. *J Natl Cancer Inst*. 2020;**112**:305-13. doi:10.1093/jnci/djz110
45. Haverkamp D, English K, Jacobs-Wingo J, et al. Effectiveness of interventions to increase colorectal cancer screening among American Indians and Alaska natives. *Prev Chronic Dis*. 2020;**17**:E62. doi:10.5888/pcd17.200049
46. O'Connor EA, Vollmer WM, Petrik AF, et al. Moderators of the effectiveness of an intervention to increase colorectal cancer screening through mailed fecal immunochemical test kits: results from a pragmatic randomized trial. *Trials*. 2020;**21**:91. doi:10.1186/s13063-019-4027-7
47. Lucas T, Thompson HS, Blessman J, et al. Effects of culturally targeted message framing on colorectal cancer screening among African Americans. *Health Psychol*. 2021;**40**:305-15. doi:10.1037/hea0001073
48. Huf SW, Asch DA, Volpp KG, et al. Text messaging and opt-out mailed outreach in colorectal cancer screening: a randomized clinical trial. *J Gen Intern Med*. 2021;**36**:1958-64. doi:10.1007/s11606-020-06415-8
49. Coronado GD, Nielson CM, Keast EM, et al. The influence of multi-morbidities on colorectal cancer screening recommendations and completion. *Cancer Causes Control*. 2021;**32**:555-65. doi:10.1007/s10552-021-01408-2
50. Shepherd ME, Lecorps A, Harris-Shapiro J, Miller-Wilson LA. Evaluating outreach methods for multi-target stool DNA test for colorectal cancer screening among an employer population. *J Prim Care Community Health*. 2021;**12**:21501327211037892. doi:10.1177/21501327211037892
51. Green BB, Anderson ML, Cook AJ, et al. A centralized program with stepped support increases adherence to colorectal cancer screening over 9 years: A randomized trial. *J Gen Intern Med*. 2022;**37**:1073-80. doi:10.1007/s11606-021-06922-2
52. Oyalowo A, Forde KA, Lamanna A, Kochman ML. Effect of patient-directed messaging on colorectal cancer screening: A randomized clinical trial. *JAMA Netw Open*. 2022;**5**:e224529. doi:10.1001/jamanetworkopen.2022.4529
53. Goshgarian G, Sorourdi C, May FP, et al. Effect of patient portal messaging before mailing fecal immunochemical test kit on colorectal cancer screening rates: A randomized clinical trial. *JAMA Netw Open*. 2022;**5**:e2146863. doi:10.1001/jamanetworkopen.2021.46863
54. Liang PS, Williams JL, Dominitz JA, et al. Age-stratified prevalence and predictors of neoplasia among U.S. adults undergoing screening colonoscopy in a national endoscopy registry. *Gastroenterol*. 2022;**163**:742-53.e4. doi: 10.1053/j.gastro.2022.05.036.
55. O'Leary MC, Reuland DS, Correa SY, et al. Uptake of colorectal cancer screening after mailed fecal immunochemical test (FIT) outreach in a newly eligible 45-49-year-old community health center population. *Cancer Causes Control*. 2023;**34**(Suppl 1):125-33. doi: 10.1007/s10552-023-01717-8.
56. Miller CA, Guidry JPD, Kenning KL, Bohl JL, Fuemmeler BF, Rivet EB. Barriers and facilitators of colorectal cancer screening during the COVID-19 pandemic. *Am Surg*. 2024;**90**:1217-23. doi: 10.1177/00031348241227216.
57. Dodd N, Carey M, Mansfield E, et al. Testing the effectiveness of a general practice intervention to improve uptake of colorectal cancer screening: A randomised controlled trial. *Aust N Z J Public Health*. 2019;**43**:464-9. doi:10.1111/1753-6405.12913
58. Hoeck S, van de Veerdonk W, De Brabander I, Kellen E. Does the Flemish colorectal cancer screening programme reach equity in FIT uptake? *Eur J Public Health*. 2019;**29**:1108-14. doi: 10.1093/eurpub/ckz043.
59. Tinmouth J, Baxter NN, Paszat LF, et al. Using physician-linked mailed invitations in an organised colorectal cancer screening programme: effectiveness and factors associated with response. *BMJ Open*. 2014;**4:**e004494. doi: 10.1136/bmjopen-2013-004494.
60. López-Kostner F, Zárate AJ, Ponce A, et al. Programa multicéntrico de cribado de cáncer colorrectal en Chile [Results of a multicentric colorectal cancer screening program in Chile]. *Rev Med Chil*. 2018;**146**:685-92. doi:10.4067/s0034-98872018000600685
61. Zhan Q, Xiang L, Zhao X, et al. Determination of withdrawal times in individualized opportunistic screening colonoscopies. *Medicine (Baltimore)*. 2019;**98**:e16819. doi:10.1097/MD.0000000000016819
62. Jørgensen OD, Kronborg O, Fenger C. A randomised study of screening for colorectal cancer using faecal occult blood testing: results after 13 years and seven biennial screening rounds. *Gut*. 2002;**50**:29-32. doi:10.1136/gut.50.1.29
63. Allam AR, Elsayed MA, Daghash IT, et al. Colonoscopy screening for colorectal cancer in Egypt: a nationwide cross-sectional study. *BMC Cancer*. 2024;**24**:131. doi: 10.1186/s12885-024-11828-3.
64. Bretthauer M, Kaminski MF, Løberg M, et al. Population-based colonoscopy screening for colorectal cancer: A randomized clinical trial. *JAMA Intern Med*. 2016;**176**:894-902. doi:10.1001/jamainternmed.2016.0960
65. Murphy N, Ward HA, Jenab M, et al. Heterogeneity of colorectal cancer risk factors by anatomical subsite in 10 European countries: A multinational cohort study. *Clin Gastroenterol Hepatol*. 2019;**17**:1323-31.e6. doi:10.1016/j.cgh.2018.07.030
66. Rat C, Pogu C, Le Donné D, et al. Effect of physician notification regarding nonadherence to colorectal cancer screening on patient participation in fecal immunochemical test cancer screening: A randomized clinical trial. *JAMA*. 2017;**318**:816-24. doi:10.1001/jama.2017.11387
67. Denis B, Ruetsch M, Strentz P, et al. Short term outcomes of the first round of a pilot colorectal cancer screening programme with guaiac based faecal occult blood test. *Gut*. 2007;**56**:1579-84. doi:10.1136/gut.2007.126037
68. Mansmann U, Crispin A, Henschel V, et al. Epidemiology and quality control of 245 000 outpatient colonoscopies. *Dtsch Arztebl Int*. 2008;**105**:434-40. doi: 10.3238/arztebl.2008.0434.
69. Brenner H, Zwink N, Ludwig L, Hoffmeister M. Should screening colonoscopy be offered from age 50? *Dtsch Arztebl Int*. 2017;**114**:94-100. doi: 10.3238/arztebl.2017.0094.
70. Hoffmeister M, Holleczek B, Stock C, et al. Utilization and determinants of follow-up colonoscopies within 6 years after screening colonoscopy: Prospective cohort study. *Int J Cancer*. 2019;**144**:402-10. doi:10.1002/ijc.31862
71. Carr PR, Weigl K, Edelmann D, et al. Estimation of absolute risk of colorectal cancer based on healthy lifestyle, genetic risk, and colonoscopy status in a population-based study. *Gastroenterol.* 2020;**159**:129-38.e9. doi: 10.1053/j.gastro.2020.03.016.
72. Gruner LF, Hoffmeister M, Ludwig L, et al. The effects of differing invitation models on the uptake of immunological fecal occult blood testing. *Dtsch Arztebl Int*. 2020;**117**:423-30. doi:10.3238/arztebl.2020.0423.
73. Heisser T, Guo F, Niedermaier T, et al. Low risk of advanced neoplasms for up to 20 years after negative colonoscopy result: Potential for personalized follow-up screening intervals. *Gastroenterol*. 2020;**159**:2235-37.e4. doi:10.1053/j.gastro.2020.08.003
74. Schwarz S, Schäfer W, Horenkamp-Sonntag D, et al. Follow-up of 3 million persons undergoing colonoscopy in Germany: Utilization of repeat colonoscopies and polypectomies within 10 years. *Clin Transl Gastroenterol*. 2020;**12**:e00279. doi: 10.14309/ctg.0000000000000279.
75. Wong MC, Ching JY, Chan VC, et al. Should prior FIT results be incorporated as an additional variable to estimate risk of colorectal neoplasia? A prospective study of 5,813 screening colonoscopies. *PLoS One*. 2014;**9**:e114332. doi:10.1371/journal.pone.0114332
76. Wong MC, Ching JY, Huang J, et al. Effectiveness of reminder strategies on cancer screening adherence: a randomised controlled trial. *Br J Gen Pract*. 2018;**68**:e604-e611. doi:10.3399/bjgp18X698369
77. Purnomo HD, Permatadewi CO, Prasetyo A, et al. Colorectal cancer screening in Semarang, Indonesia: A multicenter primary health care-based study. *PLoS One*. 2023;**18**:e0279570. doi:10.1371/journal.pone.0279570
78. Segnan N, Armaroli P, Bonelli L, et al. Once-only sigmoidoscopy in colorectal cancer screening: follow-up findings of the Italian Randomized Controlled Trial--SCORE [published correction appears in J Natl Cancer Inst. 2011 Dec 21;103(24):1903. Fracchia, M [added]]. *J Natl Cancer Inst*. 2011;**103**:1310-22. doi:10.1093/jnci/djr284
79. Yamaji Y, Mitsushima T, Ikuma H, et al. Incidence and recurrence rates of colorectal adenomas estimated by annually repeated colonoscopies on asymptomatic Japanese. *Gut*. 2004;**53**:568-72. doi:10.1136/gut.2003.026112.
80. Hoff G, Bretthauer M. Appointments timed in proximity to annual milestones and compliance with screening: randomised controlled trial. *BMJ*. 2008;**337**:a2794. doi:10.1136/bmj.a2794
81. Hoff G, Grotmol T, Skovlund E, Bretthauer M; Norwegian Colorectal Cancer Prevention Study Group. Risk of colorectal cancer seven years after flexible sigmoidoscopy screening: randomised controlled trial. *BMJ*. 2009;**338**:b1846. doi:10.1136/bmj.b1846
82. García-Albéniz X, Hsu J, Bretthauer M, Hernán MA. Effectiveness of screening colonoscopy to prevent colorectal cancer among medicare beneficiaries aged 70 to 79 years: A prospective observational study. *Ann Intern Med*. 2017;**166**:18-26. doi:10.7326/M16-0758
83. Pisera M, Franczyk R, Wieszczy P, et al. The impact of low- versus standard-volume bowel preparation on participation in primary screening colonoscopy: a randomized health services study. *Endoscopy*. 2019;**51**:227-36. doi:10.1055/a-0748-5479
84. Guiriguet C, Muñoz-Ortiz L, Burón A, et al. Alerts in electronic medical records to promote a colorectal cancer screening programme: a cluster randomised controlled trial in primary care. *Br J Gen Pract*. 2016;**66**:e483-90. doi:10.3399/bjgp16X685657
85. Valiente González L, de Miguel Ibáñez R, Escribano Sotos F. Programa piloto de cribado poblacional de cáncer colorrectal en Castilla-La Mancha. Resultados parciales tras primera ronda: 2015-2018 [Colorectal cancer screening pilot program in Castilla-La mancha. Partial results after first round: 2015-2018.]. *Rev Esp Salud Publica*. 2021;**95**:e202101011.
86. Chua AH, Koh GC. Does patient education and recommendation result in increased uptake of colorectal cancer screening using the fecal occult blood test?. *Ann Acad Med Singap*. 2014;**43**:517-18.
87. Chiu HM, Chen SL, Yen AM, et al. Effectiveness of fecal immunochemical testing in reducing colorectal cancer mortality from the One Million Taiwanese Screening Program. *Cancer*. 2015;**121**:3221-9. doi:10.1002/cncr.29462.
88. Verne JE, Aubrey R, Love SB, et al. Population based randomized study of uptake and yield of screening by flexible sigmoidoscopy compared with screening by faecal occult blood testing. *BMJ*. 1998;**317**:182-85. doi:10.1136/bmj.317.7152.182
89. Atkin WS, Hart A, Edwards R, et al. Uptake, yield of neoplasia, and adverse effects of flexible sigmoidoscopy screening. *Gut*. 1998;**42**:560-5. doi:10.1136/gut.42.4.560
90. Scholefield JH, Moss S, Sufi F, et al. Effect of faecal occult blood screening on mortality from colorectal cancer: results from a randomised controlled trial. *Gut.* 2002;**50**:840-4. doi:10.1136/gut.50.6.840.
91. Goodwin JS, Singh A, Reddy N, et al. Overuse of screening colonoscopy in the medicare population. *Arch Intern Med.* 2011;**171**:1335–43. doi:10.1001/archinternmed.2011.212
92. Morris EJ, Rutter MD, Finan PJ, et al. Post-colonoscopy colorectal cancer (PCCRC) rates vary considerably depending on the method used to calculate them: a retrospective observational population-based study of PCCRC in the English National Health Service. *Gut*. 2015;**64**:1248-56. doi:10.1136/gutjnl-2014-308362
93. Raine R, Duffy SW, Wardle J, et al. Impact of general practice endorsement on the social gradient in uptake in bowel cancer screening. *Br J Cancer*. 2016;**114**:321-26. doi:10.1038/bjc.2015.413
94. Raine R, Moss SM, von Wagner C, et al. A national cluster-randomised controlled trial to examine the effect of enhanced reminders on the socioeconomic gradient in uptake in bowel cancer screening. *Br J Cancer*. 2016;**115**:1479-86. doi:10.1038/bjc.2016.365
95. Atkin W, Wooldrage K, Parkin DM, et al. Long term effects of once-only flexible sigmoidoscopy screening after 17 years of follow-up: the UK Flexible Sigmoidoscopy Screening randomised controlled trial. *Lancet*. 2017;**389**:1299-1311. doi:10.1016/S0140-6736(17)30396-3
96. Smith SG, Wardle J, Atkin W, et al. Reducing the socioeconomic gradient in uptake of the NHS bowel cancer screening programme using a simplified supplementary information leaflet: a cluster-randomised trial. *BMC Cancer*. 2017;**17**:543. doi:10.1186/s12885-017-3512-1.
97. Costas-Muñiz R, Jandorf L, Philip E, et al. Examining the impact of latino nativity, migration, and acculturation factors on colonoscopy screening. *J Comm Health*. 2016;**41**:903-9. doi:10.1007/s10900-016-0168-8.
98. Murphy CC, Ahn C, Pruitt SL, et al. Screening initiation with FIT or colonoscopy: Post-hoc analysis of a pragmatic, randomized trial. *Prev Med*. 2019;**118**:332-35. doi:10.1016/j.ypmed.2018.11.020.
99. Miller-Wilson LA, Rutten LJF, Van Thomme J, et al. Cross-sectional adherence with the multi-target stool DNA test for colorectal cancer screening in a large, nationally insured cohort. *Int J Colorectal Dis*. 2021;**36**:2471-80. doi:10.1007/s00384-021-03956-0
100. Liu S, Wang Y, Wang Y, et al. Population-based screening for colorectal cancer in Wuhan, China. *Front Oncol*. 2024;**14**:1284975. doi: 10.3389/fonc.2024.1284975.
101. Yu Z, Li B, Zhao S, et al. Uptake and detection rate of colorectal cancer screening with colonoscopy in China: A population-based, prospective cohort study. *Int J Nurs Stud*. 2024;**153**:104728. doi: 10.1016/j.ijnurstu.2024.104728.
102. Dong X, Du L, Luo Z, et al. Combining fecal immunochemical testing and questionnaire-based risk assessment in selecting participants for colonoscopy screening in the Chinese National Colorectal Cancer Screening Programs: A population-based cohort study. *PLoS Med.* 2024;**21**:e1004340. doi: 10.1371/journal.pmed.1004340.
103. Gram MA, Therkildsen C, Clarke RB, et al. The influence of marital status and partner concordance on participation in colorectal cancer screening. *Eur J Public Health*. 2021;**31**:340-6. doi:10.1093/eurpub/ckaa206.
104. Pellat A, Deyra J, Coriat R, Chaussade S. Results of the national organised colorectal cancer screening program with FIT in Paris. *Sci Rep*. 2018;**8**:4162. doi:10.1038/s41598-018-22481-9
105. Hoffmeister M, Holleczek B, Zwink N, et al. Screening for bowel cancer: Increasing participation via personal invitation. *Dtsch Arztebl Int*. 2017;**114**:87-93. doi:10.3238/arztebl.2017.0087
106. Zorzi M, Grazzini G, Senore C, Vettorazzi M. Screening for colorectal cancer in Italy: 2004 survey. *Epidemiol Prev*. 2006;**30**:41-50.
107. Zorzi M, Baracco S, Fedato C, et al. Screening for colorectal cancer in Italy, 2009 survey. *Epidemiol Prev*. 2011;**35**:55-77.
108. Zorzi M, Fedato C, Grazzini G, et al. Lo screening colorettale in Italia, survey 2010 [Screening for colorectal cancer in Italy, 2010 survey]. *Epidemiol Prev*. 2012;**36**:55-77.
109. Zorzi M, Da Re F, Mantellini P, Naldoni C, Sassoli De'Bianchi P, Senore C, Turrin A, Visioli CB, Zappa M; Italian colorectal cancer screening survey group. Screening for colorectal cancer in Italy: 2011-2012 survey. *Epidemiol Prev.* 2015;**39**:93-107.
110. Saito H, Ozaki A, Murakami M, et al. The long term participation trend for the colorectal cancer screening after the 2011 triple disaster in Minamisoma City, Fukushima, Japan. *Sci Rep*. 2021;**11**:23851. doi:10.1038/s41598-021-03225-8
111. Rim JH, Youk T, Kang JG, et al. fecal occult blood test results of the national colorectal cancer screening program in South Korea (2006-2013). *Sci Rep*. 2017**;7**:2804. doi:10.1038/s41598-017-03134-9.
112. Santare D, Kojalo I, Huttunen T, et al. Improving uptake of screening for colorectal cancer: a study on invitation strategies and different test kit use. *Eur J Gastroenterol Hepatol*. 2015;**27**:536-43. doi:10.1097/MEG.0000000000000314.
113. Dulskas A, Poskus T, Kildusiene I, et al. National colorectal cancer screening program in Lithuania: Description of the 5-year performance on population level. *Cancers (Basel)*. 2021;**13**:1129. doi:10.3390/cancers13051129.
114. Tamin NSI, Razalli KA, Sallahuddin SN, et al. A 5-year evaluation of using stool-based test for opportunistic colorectal cancer screening in primary health institutions across Malaysia. *Cancer Epidemiol*. 2020;**69**:101829. doi:10.1016/j.canep.2020.101829.
115. Quyn AJ, Fraser CG, Stanners G, et al. Uptake trends in the Scottish Bowel Screening Programme and the influences of age, sex, and deprivation. *J Med Screen*. 2018;**25**:24-31. doi:10.1177/0969141317694065.
116. Cheng SY, Chen CF, He HC, et al. Impact of COVID-19 pandemic on fecal immunochemical test screening uptake and compliance to diagnostic colonoscopy. *J Gastroenterol Hepatol*. 2021;**36**:1614-19. doi:10.1111/jgh.15325.
117. UK Colorectal Cancer Screening Pilot Group. Results of the first round of a demonstration pilot of screening for colorectal cancer in the United Kingdom. *BMJ*. 2004;**329**:133. doi:10.1136/bmj.38153.491887.7C
118. Steele RJ, Kostourou I, McClements P, et al. Effect of repeated invitations on uptake of colorectal cancer screening using faecal occult blood testing: analysis of prevalence and incidence screening. *BMJ*. 2010;**341**:c5531. doi:10.1136/bmj.c5531.
119. Lo SH, Vart G, Snowball J, et al. The impact of media coverage of the Flexible Sigmoidoscopy Trial on English colorectal screening uptake. *J Med Screen*. 2012;**19**:83-8. doi:10.1258/jms.2012.012017.
120. Logan RF, Patnick J, Nickerson C, et al. Outcomes of the Bowel Cancer Screening Programme (BCSP) in England after the first 1 million tests. *Gut*. 2012;**61**:1439-46. doi:10.1136/gutjnl-2011-300843.
121. Lo SH, Halloran S, Snowball J, et al. Colorectal cancer screening uptake over three biennial invitation rounds in the English bowel cancer screening programme. *Gut.* 2015;**64**:282-91. doi:10.1136/gutjnl-2013-306144.
122. Kaminski MF, Bretthauer M, Zauber AG, et al. The NordICC Study: rationale and design of a randomized trial on colonoscopy screening for colorectal cancer. *Endoscopy*. 2012;**44**:695-702. doi:10.1055/s-0032-1306895
123. Kaminski MF, Kraszewska E, Rupinski M, et al. Design of the Polish Colonoscopy Screening Program: a randomized health services study. *Endoscopy*. 2015;**47**:1144-50. doi:10.1055/s-0034-1392769
124. Hirst Y, Kerrison R, Kobayashi LC, et al. Text Reminders in Colorectal Cancer Screening (TRICCS): Protocol for a randomised controlled trial. *BMC Public Health*. 2016;**16**:74. doi:10.1186/s12889-016-2733-6
125. Cooper GS, Kou TD, Schluchter MD, et al. Changes in receipt of cancer screening in Medicare beneficiaries following the Affordable Care Act. *J Natl Cancer Inst*. 2015;**108**:djv374. doi:10.1093/jnci/djv374.
126. Doubeni CA, Laiyemo AO, Reed G, et al. Socioeconomic and racial patterns of colorectal cancer screening among Medicare enrollees in 2000 to 2005. *Cancer Epidemiol Biomarkers Prev*. 2009;**18**:2170-5. doi:10.1158/1055-9965.EPI-09-0104.
127. Tak HJ, Pan IW, Halpern MT, Shih YT. Impact of race-specific screening guideline on the uptake of colorectal cancer screening among young African Americans. *Cancer Med*. 2022;**11**:5013-24. doi:10.1002/cam4.4842.
128. Liu PH, Sanford NN, Liang PS, Singal AG, Murphy CC. Persistent Disparities in Colorectal Cancer Screening: A Tell-Tale Sign for Implementing New Guidelines in Younger Adults. *Cancer Epidemiol Biomarkers Prev.* 2022;**31**:1701-1709. doi: 10.1158/1055-9965.EPI-21-1330.
129. Shim JI, Kim Y, Han MA, et al. Results of colorectal cancer screening of the national cancer screening program in Korea, 2008. *Cancer Res Treat*. 2010;**42**:191-8. doi:10.4143/crt.2010.42.4.191.
130. Koo JH, Leong RW, Ching J, et al. Knowledge of, attitudes toward, and barriers to participation of colorectal cancer screening tests in the Asia-Pacific region: a multicenter study. *Gastrointest Endosc*. 2012;**76**:126-35. doi:10.1016/j.gie.2012.03.168
131. Ansa BE, Datta B, Ibrahim S, Islam KMM, Saucier A, Coffin J. Role of Social Support in Screening Colonoscopy/Sigmoidoscopy Uptake among U.S. Adults. *Healthcare (Basel).* 2024;**12**:344. doi: 10.3390/healthcare12030344.
132. Walsh JM, Kaplan CP, Nguyen B, et al. Barriers to colorectal cancer screening in Latino and Vietnamese Americans. Compared with non-Latino white Americans. *J Gen Intern Med*. 2004;**19**:156-66. doi:10.1111/j.1525-1497.2004.30263.x.
133. Ko CW, Kreuter W, Baldwin LM. Persistent demographic differences in colorectal cancer screening utilization despite Medicare reimbursement. *BMC Gastroenterol*. 2005;**5**:10. doi:10.1186/1471-230X-5-10.
134. Thompson B, Coronado G, Neuhouser M, Chen L. Colorectal carcinoma screening among Hispanics and non-Hispanic whites in a rural setting. *Cancer.* 2005;**103**:2491-8. doi:10.1002/cncr.21124.
135. Farmer MM, Bastani R, Kwan L, et al. Predictors of colorectal cancer screening from patients enrolled in a managed care health plan. *Cancer*. 2008;**112**:1230-8. doi:10.1002/cncr.23290.
136. McNeill LH, Coeling M, Puleo E, et al. Colorectal cancer prevention for low-income, sociodemographically-diverse adults in public housing: baseline findings of a randomized controlled trial. *BMC Public Health*. 2009;**9**:353. doi:10.1186/1471-2458-9-353.
137. Walter LC, Lindquist K, Nugent S, et al. Impact of age and comorbidity on colorectal cancer screening among older veterans. *Ann Intern Med*. 2009;**150**:465-73. doi:10.7326/0003-4819-150-7-200904070-00006.
138. Maxwell AE, Crespi CM. Trends in colorectal cancer screening utilization among ethnic groups in California: are we closing the gap? *Cancer Epidemiol Biomarkers Prev*. 2009;**18**:752-9. doi:10.1158/1055-9965.EPI-08-0608.
139. Adams-Campbell LL, Makambi K, Mouton CP, et al. Colonoscopy utilization in the Black Women's Health Study. *J Natl Med Assoc*. 2010;**102**:237-42. doi:10.1016/s0027-9684(15)30530-7.
140. Cokkinides V, Bandi P, Shah M, et al. The association between state mandates of colorectal cancer screening coverage and colorectal cancer screening utilization among US adults aged 50 to 64 years with health insurance. *BMC Health Serv Res*. 2011;**11**:19. doi:10.1186/1472-6963-11-19.
141. Day LW, Espey DK, Madden E, et al. Screening prevalence and incidence of colorectal cancer among American Indian/Alaskan natives in the Indian Health Service. *Dig Dis Sci.* 2011;**56**:2104-13. doi:10.1007/s10620-010-1528-3.
142. Khatami S, Xuan L, Roman R, et al. Modestly increased use of colonoscopy when copayments are waived. *Clin Gastroenterol Hepatol.* 2012;**10**:761-66.e1. doi:10.1016/j.cgh.2012.02.027.
143. Sinicrope PS, Goode EL, Limburg PJ, et al. A population-based study of prevalence and adherence trends in average risk colorectal cancer screening, 1997 to 2008. *Cancer Epidemiol Biomarkers Prev*. 2012;**21**:347-50. doi:10.1158/1055-9965.EPI-11-0818.
144. Doubeni CA, Jambaulikar GD, Fouayzi H, et al. Neighborhood socioeconomic status and use of colonoscopy in an insured population--a retrospective cohort study. *PLoS One.* 2012;**7**:e36392. doi:10.1371/journal.pone.0036392.
145. Cohen SS, Murff HJ, Signorello LB, Blot WJ. Obesity and colorectal cancer screening among black and white adults. *Cancer Causes Control*. 2012;**23**:709-16. doi:10.1007/s10552-012-9940-y.
146. May FP, Bromley EG, Reid MW, et al. Low uptake of colorectal cancer screening among African Americans in an integrated Veterans Affairs health care network*. Gastrointest Endosc*. 2014;**80**:291-8. doi:10.1016/j.gie.2014.01.045.
147. Halpern MT, Romaire MA, Haber SG, et al. Impact of state-specific Medicaid reimbursement and eligibility policies on receipt of cancer screening. *Cancer*. 2014;**120**:3016-24. doi:10.1002/cncr.28704.
148. Wilcox ML, Acuña JM, de la Vega PR, et al. Factors affecting compliance with colorectal cancer screening among households residing in the largely Haitian community of Little Haiti, Miami-Dade County, Florida: an observational study. *Medicine (Baltimore).* 2015;**94**:e806. doi:10.1097/MD.0000000000000806.
149. Kotwal AA, Lauderdale DS, Waite LJ, Dale W. Differences between husbands and wives in colonoscopy use: Results from a national sample of married couples. *Prev Med*. 2016;**88**:46-52. doi:10.1016/j.ypmed.2016.03.011.
150. Richman I, Asch SM, Bhattacharya J, Owens DK. Colorectal cancer screening in the era of the Affordable Care Act. *J Gen Intern Med*. 2016;**31**:315-20. doi:10.1007/s11606-015-3504-2.
151. Waghray A, Jain A, Waghray N. Colorectal cancer screening in African Americans: practice patterns in the United States. Are we doing enough? *Gastroenterol Rep (Oxf).* 2016;**4**:136-40. doi:10.1093/gastro/gow005.
152. Siantz E, Wu B, Shiroishi M, et al. Mental illness is not associated with adherence to colorectal cancer screening: Results from the California Health Interview Survey. *Dig Dis Sci.* 2017;**62**:224-34. doi:10.1007/s10620-016-4366-0.
153. Ghai NR, Jensen CD, Corley DA, et al. Colorectal cancer screening participation among Asian Americans overall and subgroups in an integrated health care setting with organized screening. *Clin Transl Gastroenterol*. 2018;**9**:186. doi:10.1038/s41424-018-0051-2.
154. Buehler JW, Castro JC, Cohen S, et al. Personal and neighborhood attributes associated with cervical and colorectal cancer screening in an urban African American population. *Prev Chronic Dis*. 2019;**16**:E118. doi:10.5888/pcd16.190030.
155. Bhandari NR, Li C. Impact of the Affordable Care Act's elimination of cost-sharing on the guideline-concordant utilization of cancer preventive screenings in the United States Using Medical Expenditure Panel Survey. *Healthcare (Basel).* 2019;**7**:36. doi:10.3390/healthcare7010036.
156. Rastogi N, Xia Y, Inadomi JM, et al. Disparities in colorectal cancer screening in New York City: An analysis of the 2014 NYC Community Health Survey. *Cancer Med.* 2019;**8**:2572-9. doi:10.1002/cam4.2084.
157. Viramontes O, Bastani R, Yang L, et al. Colorectal cancer screening among Hispanics in the United States: Disparities, modalities, predictors, and regional variation. *Prev Med*. 2020;**138**:106146. doi:10.1016/j.ypmed.2020.106146.
158. Alyabsi M, Meza J, Islam KMM, et al. Colorectal Cancer Screening Uptake: Differences Between Rural and Urban Privately-Insured Population. *Front Public Health.* 2020;**8**:532950. doi:10.3389/fpubh.2020.532950.
159. Ng S, Xia Y, Glenn M, et al. Factors associated with up-to-date colonoscopy use among Puerto Ricans in New York City, 2003-2016. *Dig Dis Sci.* 2021;**66**:2907-15. doi:10.1007/s10620-020-06648-x.
160. McEvoy CS, Shah NG, Roberts SE, et al. Universal healthcare coverage does not ensure adherence to initial colorectal cancer screening guidelines. *Mil Med*. 2021;**186**:e1071-6. doi:10.1093/milmed/usaa319.
161. Ansa BE, Lewis N, Hoffman Z, et al. Evaluation of blood stool test utilization for colorectal cancer screening in Georgia, USA. *Healthcare (Basel)*. 2021;**9**:569. doi:10.3390/healthcare9050569.
162. Fisher DA, Princic N, Miller-Wilson LA, et al. Utilization of a colorectal cancer screening test among individuals with average risk. *JAMA Netw Open*. 2021;**4**:e2122269. doi:10.1001/jamanetworkopen.2021.22269.
163. Fisher DA, Princic N, Miller-Wilson LA, et al. Healthcare costs of colorectal cancer screening and events following colonoscopy among commercially insured average-risk adults in the United States. *Curr Med Res Opin*. 2022;**38**:427-34. doi:10.1080/03007995.2021.2015157.
164. Kowalkowski H, Austin G, Guo Y, Miller-Wilson LA, DaCosta Byfield S. Patterns of colorectal cancer screening and adherence rates among an average-risk population enrolled in a national health insurance provider during 2009-2018 in the United States. *Prev Med Rep*. 2023;**36**:102497. doi: 10.1016/j.pmedr.2023.102497.
165. Hermann S, Friedrich S, Haug U, et al. Association between socioeconomic and demographic characteristics and utilization of colonoscopy in the EPIC-Heidelberg cohort. *Eur J Cancer Prev*. 2015;**24**:81-8. doi:10.1097/CEJ.0000000000000080.
166. Mohammad BF, Andsoy II. Health behaviors, knowledge, screening, and attitudes toward colorectal cancer among Iraqi adults. *Public Health Nurs*. 2024;**41**:514-24. doi: 10.1111/phn.13302.
167. Sharma A, Alatise OI, O'Connell K, et al. Healthcare utilisation, cancer screening and potential barriers to accessing cancer care in rural South West Nigeria: a cross-sectional study. *BMJ Open*. 2021;**11**:e040352. doi:10.1136/bmjopen-2020-040352.
168. Thulin T, Strömberg U, Holmén A, et al. Sociodemographic changes in the population frequency of colonoscopy following the implementation of organised bowel cancer screening: An analysis of data from Swedish registers, 2006-2015. *J Med Screen*. 2021;**28**:244-51. doi:10.1177/0969141320957708.
